# Temporal and spatial niche partitioning in a retrotransposon community of the *Drosophila melanogaster* genome

**DOI:** 10.1093/nar/gkaf516

**Published:** 2025-06-12

**Authors:** Marion Varoqui, Mourdas Mohamed, Bruno Mugat, Daniel Gourion, Maëlys Lemoine, Alain Pélisson, Charlotte Grimaud, Séverine Chambeyron

**Affiliations:** Institute of Human Genetics, CNRS, University of Montpellier, 34396 Montpellier, France; Institute of Human Genetics, CNRS, University of Montpellier, 34396 Montpellier, France; Institute of Human Genetics, CNRS, University of Montpellier, 34396 Montpellier, France; Avignon Université, LMA UPR 2151, Avignon 84000, France; Institute of Human Genetics, CNRS, University of Montpellier, 34396 Montpellier, France; Institute of Human Genetics, CNRS, University of Montpellier, 34396 Montpellier, France; Institute of Human Genetics, CNRS, University of Montpellier, 34396 Montpellier, France; Institute of Human Genetics, CNRS, University of Montpellier, 34396 Montpellier, France

## Abstract

Transposable elements (TEs) are genetic parasites that can potentially threaten the stability of the genomes they colonize. Nonetheless, TEs persist within genomes and are rarely fully eliminated, with diverse TE families coexisting in varing copy numbers. The TE replication strategies that enable host organisms to tolerate and accommodate the extensive diversity of TEs, while minimizing harm to the host and avoiding mutual competition among TEs, remain poorly understood. Here, by studying the spontaneous or experimental mobilization of four *Drosophila* LTR RetroTransposable Elements (LTR-RTEs), we reveal that each of them preferentially targets open chromatin regions characterized by specific epigenetic features. Among these, gtwin and ZAM are expressed in distinct cell types within female somatic gonadal tissues and inserted into the distinct accessible chromatin landscapes of the corresponding stages of embryogenesis. These findings suggest that individual LTR-RTEs exploit unique biological niches, enabling their coexistence within the tightly regulated ecosystem of the same host genome.

## Graphical Abstract

**Graphical Abstract:**
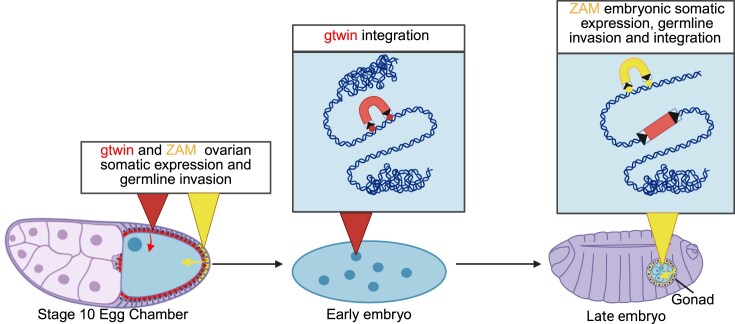

## Introduction

Proper development of multicellular organisms relies on the temporally and spatially regulated expression of genes encoded by the genome. However, not every DNA sequence, even if it can be expressed within a genome, contributes to the fitness of the organism. Some sequences, such as transposable elements (TEs) exhibit a self-serving behavior due to their ability to take advantage of the host proteins to favor their expression and transposition in various locations within the host genome. Thus, they can be considered as genomic parasites [1]. This capacity to move within the genome can generate harmful mutations such as disruptions of coding sequences, impairment of gene regulation, and chromosomal rearrangements by ectopic recombination [2], which may ultimately jeopardize the integrity of the host genome. This is particularly relevant considering that maintenance of TEs in a host organism throughout generations requires that they reach the germline, the carrier of the heritable host genetic information. In this context, the key question is to determine how each TE has been able to tune up its replication cycle in order to prevent extinction of either the host or the TE [3]. On the other hand, during evolution, several defense mechanisms have been developed by the host to keep replicative transposition rates at low levels allowing proper balance between host survival and TE maintenance. Some of the mechanisms affecting the level of TE transposition involve, in the germline and surrounding somatic cells, a specific class of small regulatory RNAs known as Piwi-interacting RNAs (piRNAs), which, when associated with PIWI proteins, a subclass of Argonaute proteins, can hybridize with nascent or cytoplasmic TE transcripts. This specific targeting by the host defense machinery leads to the silencing of TEs, either transcriptionally (TGS) or post-transcriptionally (PTGS), respectively [4–7]. We assume that present-day TE landscapes likely result from evolution of a series of host–TE interactions, including transposition repression, that, together, prevented extinction of either the host or the TE [3, 8]. On the TE–host side, eukaryotic genomes could be considered as ecosystems differing in the amount and diversity of the TEs they harbor [9–11]. Following this idea, we hypothesize that the coexistence of numerous TE families within the same host genome was made possible by the evolution of specific features in the replication cycle of each family, which has allowed them to persist without harming the host and/or competing with each other.

As much as 20% of the *Drosophila melanogaster* genomic sequences derive from different classes of TEs [12]. Within the same class, comparative analyses of conserved TE-encoding proteins have allowed their distribution into specific clades, each segregated into distinct families. For instance, comparative analyses of the conserved reverse transcriptase domain of Long Terminal Repeat-RetroTransposable Elements (LTR-RTEs), a class replicating via an RNA intermediate and representing ∼10% of the *D. melanogaster* genome [12], revealed a distribution into three clades: Copia, BEL, and Gypsy [13, 14]. LTR-RTEs of the Copia and BEL clades encode a single open reading frame (ORF) and are represented by a few families. The much more expanded Gypsy clade, on the other hand, displays a stronger heterogeneity in coding sequence with LTR-RTE families encoding one, two, or three ORFs [13, 14]. Several studies, using various *Drosophila* strains, have revealed that the Gypsy clade families are not only genetically diversified but have also adopted distinct niches of expression. While some families within this clade are expressed in germline cells, like most TEs, the majority are specifically expressed in the somatic cells surrounding the germline [15]. This unique replication strategy is linked to the acquisition, by their common ancestor, of an ORF encoding a viral-like envelope protein which enables these elements to infect the germline [13, 15]. By uncoupling the site of expression (tolerant, differentiated gonadal somatic cells) from the site of integration (germline), this replication cycle is likely less harmful for the host germline, providing a possible explanation for the evolutionary success of this clade. Moreover, the high diversity existing between the gonadal somatic cell types that are potential sources of viral-like particles able to infect the germline has been fully exploited for the wide evolutionary diversification of this clade. Indeed, each type of ovarian somatic cell seems to be adapted as a specific niche for the expression of a particular LTR-RTE family [15]. This expression niche partitioning probably provided diverse unique environments where families can thrive without having to compete with each other.

Similarly, the integration of TEs in eukaryotic genomes seems not random, indicating that several host–TE interactions have certainly been developed to allow such genome partitioning of TE insertions. Pioneering studies in the budding yeast *Saccharomyces cerevisiae* have revealed how co-optation of distinct endogenous proteins as TE cofactors have notably driven insertion niche partitioning for several LTR-RTEs families. Indeed, it appears that the LTR-RTE Ty5 benefits from the interaction of its integrase with the heterochromatin protein Sir4 to preferentially integrate in subtelomeric regions. On the other hand, two distinct LTR-RTEs Ty1 and Ty3 rather integrate in nonessential multicopy genes transcribed by RNA PolIII such as tranfer RNAs (tRNAs) genes [16]. Note, although they share the same global insertion environment, each of these elements has its own insertion site preference that is mainly dependent on the interaction of its integrase with specific cellular cofactors [16].

More recent data in *D. melanogaster* suggest that TEs, which belong to distinct classes and differ by their transposition mechanism, generally share insertion preference for open chromatin regions [17, 18], but display distinct insertion patterns. For example, the DNA transposon P-element favors integration of its DNA in replication origins [17, 19], while RTEs integrate their complematary DNAs (cDNAs) either near the promoters and exons of active genes, for all LTR-RTEs families, or toward the telomere, for the non-LTR-RTE I-element [17]. Whether integration preferences may also vary between the different families of the same LTR class and/or are influenced by specific cellular contexts are still open questions.

Studying LTR-RTE ecology regarding not only the interactions between a TE family and its host but also between members of the whole community of TE families having colonized the same host is, therefore, expected to provide further insights into the ways they have successfully invaded all present-day eukaryotic genomes.

To investigate to what extent the active LTR-RTE families present in a same organism may differ in their replication cycles, we used a *D. melanogaster* strain that we had previously constructed to impair the Piwi-mediated LTR-RTE repression specifically in the somatic tissue of the gonads (Supplementary Fig. S1) [20]. This strain contains a traffic-jam-Gal4/Gal80^ts^ inducible driver which activates, at permissive temperature, the expression of a short RNA hairpin targeting Piwi (sh-piwi) in the gonadal somatic cells. This somatic knockdown (sKD) alleviates LTR-RTE repression in these cells without causing sterility [20]. When females of this strain are transferred for 5 days from the 20°C nonpermissive to the 25°C permissive temperature, they display a partial depletion of the Piwi protein in their ovarian somatic cells (piwi-sKD), leading to an accumulation of LTR-RTE transcripts in these cells. *De novo* germline insertions of two LTR-RTEs from the Gypsy clade, ZAM and gtwin, were detected in short-read genomic libraries from embryos sequenced two generations (F2) after piwi-sKD (Supplementary Fig. S1) [20]. As a proof of concept, we also demonstrated that this strain is a powerful tool for studying the accumulation of *de novo* germline insertions of at least ZAM and gtwin LTR-RTEs. At that time, it was illustrated by the increase in their copy number, approximately estimated by genomic polymerase chain reaction (PCR), following the application of piwi-sKD through successive generations up to generation 72.

In the present study, by performing long-read sequencing of genomic DNA obtained from F2 male flies after 11, 31, and 73 generations of piwi-sKD, we were able to (i) verify the increased numbers of new insertions for ZAM and gtwin across successive generations and also document new insertions for three other LTR-RTEs families namely roo, copia, and rover; and (ii) map enough new germinal insertions of four of these LTR-RTE families to reveal differences in their landing site preferences, particularly for distinct epigenetic marks associated with open chromatin. Furthermore, we highlighted that gtwin and ZAM replication cycles exhibit differences not only in their expression patterns but also in the timing of their integration into the different accessible chromatin landscapes of the developing embryonic germline, which could explain some of their site preferences. Our findings emphasize how, over the course of evolution, the diversity of the cell identities that different LTR-RTE families exploit for both expression and integration has facilitated their colonization of specific niches, enabling their coexistence within this ecosystem.

## Materials and methods

### *Drosophila*genotypes

**Figure 1.**
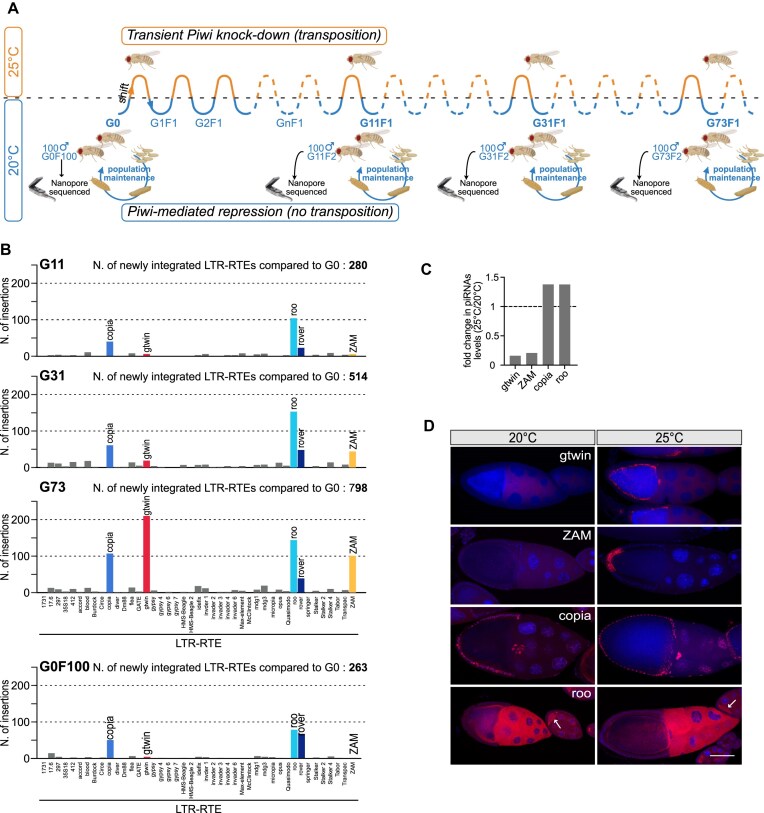
Four LTR-RTEs exhibit transcriptional and insertional activity across successive generations. (**A**) Schematic representation of the temperature change from 20°C to 25°C applied to adult flies for 5 days at each generation to induce a transient somatic knockdown of Piwi (Piwi-sKD) followed by constant maintenance at 20°C until the next generation. G0F100 corresponds to a sub-population of the initial G0 parental strain that was constantly kept at 20°C for 100 generations. During the successive Piwi-sKDs, three large populations corresponding to the offsprings of flies at generations 11, 31, and 73 (G11F1, G31F1, and G73F1) were isolated and kept at 20°C. The offsprings of these isolated populations (G11F2, G31F2, and G73F2) were raised at 20°C and were sequenced using Nanopore technology. Created in BioRender. MUGAT, B. (2025) https://BioRender.com/9rpicr9. (**B**) Quantification of the new LTR-RTE insertions annotated in G11F2, G31F2, and G73F2 and in the control population G0F100, as compared to the initial parental G0 strain. Total number of newly integrated LTR-RTEs is indicated for each generation in bold at the top of each chart. (**C**) Bar plots showing the fold change in antisense piRNA reads, normalized to the total piRNA reads, in Piwi-sKD ovaries (25°C) compared to control ovaries (20°C) for the four LTR-RTEs (gtwin, ZAM, copia, and roo). (**D**) Representative images of stage 10 ovarian expression patterns obtained for gtwin, ZAM, copia, and roo LTR-RTEs by smiFISH (in red) at the nonpermissive temperature, 20°C, or after 5 days at the permissive temperature, 25°C (Piwi-sKD). DNA was stained with 4,6-diamidino-2-phenylindole (DAPI; blue). Bar represents 50 μm.

As previously described (Supplementary Fig. S1) [20], all flies used to determine LTR-RTE mobilization and integration, shared the genotype of the founder G0 strain : *w; tj-Gal4; tubP-Gal80^ts^*, *sh-piwi*. The polymorphism of this strain had been partially reduced by isolating a single pair of parents, and the strain was thereafter stably maintained at 20°C as a large population (>500 progenitors at each of the 100 successive generations of the G0F100 population). An independent subset of the G0 population was bred using >500 flies per generation, the temperature being raised at each generation from 20°C to 25°C for a 5-day period during the adult stage (Fig. 1A). At the 11th (G11), 31th (G31), and 73rd (G73) generation, a large subset of GnF1 progenitors (∼500 flies, from the *n*th generation of interest) of the treated population was isolated and maintained *en masse* at 20°C, the nonpermissive temperature for piwi-sKD. A strain harboring the genotype: *w*; *vas::EGFP* was also used [21]. The *piwi-AID-GFP* strain was a generous gift of G. Hannon [22].

### Oxford nanopore technology (ONT) sequencing data analysis

As previously described [23], genomic DNA was extracted from 100 GnF2 males (Fig. 1A), and long-read sequencing data were analyzed using the TrEMOLO software (v2.2) [24] with some modifications. To detect newly integrated TEs, we employed the OUTSIDER TE detection module with, as a reference, the Dmel_R6.32 reference genome from FlyBase (v.104). Settings parameters for size and identity were set at 80%. The LTR-RTE database was extracted from the collection of reference TEs from Bergman’s laboratory (https://github.com/bergmanlab/transposons). The quality of the reads is presented in Supplementary Table S1. According to [24], the sequencing depths of all libraries (except for that of the nonpolymorphic G0 strain) were estimated to be similar enough to spare us from down sampling the largest ones (Supplementary Table S1). Frequency estimation was conducted using the TE analysis module of TrEMOLO (v2.5), and reads identified as clipped reads by TrEMOLO were excluded from the frequency calculation.

### Annotation of false positive new insertions

The G0F100 library and the other libraries were respectively established with two populations that independently evolved from a shared G0 ancestor strain. Consequently, any insertion found in both the G0F100 and any other library was attributed to the G0 parental genome. This allowed us to annotate as false negative pre-existing insertions those that were likely missed in the low quality G0 parental library, characterized by low coverage and shorter reads. All annotations were performed on the Dmel_R6.32 reference genome from FlyBase (v.104), which contains both annotated euchromatic and mapped heterochromatic regions for all chromosomes (2L, 2R, 3L, 3R, 4, and X). Highly repetitive and unlocalized heterochromatic regions, such as large satellite blocks, were excluded from our analyses due to their ambiguous mapping, which prevents reliable insertion site annotation.

### Annotation of newly integrated LTR-RTEs in piRNA clusters

The piRNA clusters were annotated on the Dmel_R6.32 reference genome using the published database https://www.smallrnagroup.uni-mainz.de/piRNAclusterDB/data/FASTA/Drosophila_melanogaster.piRNAclusters.gtf ). Then a comparison between piRNA cluster coordinates and the LTR-RTE coordinates was used to determine the presence of new insertion in piRNA clusters.

### Small RNA purification and sequencing

Small RNAs from ovaries collected at permissive (25°C) and nonpermissive (20°C) temperature, for Piwi-sKD, were isolated using TraPR ion exchange spin columns (Lexogen, Catalog Nr.128.08). The libraries were performed by MGX-Biocampus Montpellier platform using the NEBNext^®^ Small RNA Library Prep Set for Illumina^®^ from NEB. The sequencing was performed on flow cell SP paired-end 28–90 nt on NOVASEQ 6000 apparatus by MGX. Raw reads were trimmed from their 3′ linkers and loaded on a homemade pipeline available at https://bitbucket.org/blaiseli/pirna-pipeline previously used [20]. Briefly, trimmed reads (18–30 nts in size) were mapped with Bowtie2 [25] using mismatch-tolerant settings to the *D. melanogaster* genome (release 5; dm3) complemented with canonical TEs (*Drosophila* consensus TE sequences taken from https://github.com/cbergman/transposons). Reads were annotated based on their mapping coordinates. Small RNAs mapping on piRNA clusters [26], ovary siRNA clusters [27], TEs or 3′UTR of coding genes (ftp://ftp.flybase.net/), and not to rRNAs or miRNAs were defined. Candidate piRNAs were a subset of the above defined reads with a size between 23 and 30 nucleotides. Candidate piRNAs were mapped again on canonical TE sequences. Data were normalized using the total of piRNA reads.

### Single-molecule inexpensive RNA fluorescence *in situ* hybridization probe preparation

39–48 probes of 20 nucleotides targeting specifically ZAM, gtwin, roo, or copia transcripts were designed using Oligostan script [28]. Primary probes were produced in 96-well plates. For convenience, the oligonucleotides are delivered in Tris-EDTA pH 8.0 (TE) buffer, at final concentration of 100 μM. An equimolar mixture of the different primary probes was prepared and diluted five times in TE buffer to obtain a final concentration of 0.833 μM for each individual probe. Fluorescent-labeled FLAP-X (5′-Cy3/CACT GAG TCC AGC TCG AAA CTT AGG AGG/Cy3-3′) or FLAP-Y (5′-Cy3/AA TGC ATG TCG ACG AGG TCC GAG TGT AA/Cy3-3′) was delivered lyophilized and resuspended in TE buffer at final concentration of 100 μM. The reverse complement of each of these respective sequences was added at the 3′end of each specific probe (Supplementary Table S7). Annealing between specific probes and their respective FLAP was performed as previously described [28] and then diluted in hybridization buffer.

### SmiFISH in ovaries and embryos

Ovaries were dissected in phosphate-buffered saline (PBS)1X and fixed during 20 min in PBS-Triton 0.3% (PBS-Tr) containing 4% formaldehyde. After several washes in PBS-Tr, ovaries were immersed in 100% methanol by successive baths in a PBS-Tr solution containing an increasing percentage of methanol. At this stage, ovaries can be kept in methanol at −20°C for several weeks. Embryos were collected and dechorionated in 2.6% bleach. They were rinsed extensively with water and fixed in 1:1 volume of fixative solution (4% formaldehyde, 60 mM KCl, 150 mM NaCl, 0.5 mM spermidine, 0.15 mM spermine, 2 mM EDTA, 0.5 mM EGTA, and 15 mM PIPES) and heptane for 25 min at room temperature with agitation. Upon removal of the aqueous phase, an equal volume of 100% methanol was added before being vortexed for 1 min. Devitellinized embryos were collected from the methanol phase and then washed three times with 100% methanol. At this stage, embryos can be kept in methanol at −20°C for several weeks. Fixed embryos or ovaries were first washed twice in 50% methanol/50% ethanol for 5 min, rinsed twice in 100% ethanol, and then washed two times in 100% ethanol for 5 min. They were incubated in blocking buffer (PBS 1×, Tween 0.1%, RNAsin, and BSA 0.2 mg/ml in nuclease-free H_2_O) for 1 h (a wash every 15 min) and once in wash buffer (2× SSC, deionized formamide 10%, and H_2_O in nuclease-free H_2_O) before the O/N incubation at 37°C at 350 rpm with smiFISH probes (Supplementary Table S6) and either an anti-Rat Vasa antibody (DHSB, 1:120) or a Guinea Pig traffic jam antibody (gift from D. Godt [29], Toronto, 1: 120) diluted in the hybridization buffer [10% deionized formamide, 2× SSC, 100 mg tRNA, 5% dextran sulfate, 2 mM vanadyl ribonucleoside complex (VRC) (NEB), and 0.2 mg/ml BSA]. Subsequently, embryos/ovaries were washed with a wash buffer twice for 1 h at 37°C and once for 1 h at room temperature. Embryos were transferred in PBS, 0.1% Tween (PBT), and 10% donkey serum, and either Donkey anti Rat Alexa 488 (Molecular Probes) or Donkey anti Guinea Pig Alexa 594 (Molecular Probes) was added at 1:500 dilution. After several washes in PBT and 4,6-diamidino-2-phenylindole (DAPI) staining, embryos/ovaries were mounted in ProLong Gold Antifade mounting medium (Thermo Fisher Scientific). smiFISH coupled with vasa immunostaining was imaged with LSM 880 with SR Airyscan module (Zeiss) using ×40/1.4 NA objective. Airyscan processing was performed using 2D Zen Black v3.2 (Zeiss) prior to analysis. smiFISH coupled with traffic jam immunostaining was imaged with Leica SP8 confocal microscope equipped with ×40/1.4 NA objectives. Image acquisition was done with the following settings: 2048 × 2048 pixels or 1024 × 1024 pixels, 16-bit depth.

### Immunofluorescence on *Drosophila* embryos

We performed double immunostaining on fixed embryos, from a cross between females of the G73 population and males expressing Piwi coupled to GFP [22], with a mouse anti-traffic jam antibody (M. Siomi, NIG-Fly) and a rabbit anti-GFP antibody (Abcam, ab290). Fixed embryos stored in 100% methanol were successively incubated during 15 min in 90/10, 70/30, 50/50, and 30/70 percent methanol/PBT. They were permeabilized with PBS-Tr for 30 min and blocked in a PBS-Tr solution containing 10% NDS (Normal Donkey Serum) for 1 h. Embryos were incubated overnight at 4°C on a rotating wheel with PBSTr-10% NDS containing the two primary antibodies diluted 1:500. After several washes in PBS-Tr, embryos were incubated during 45 min at room temperature with PBS-Tr-10%NDS containing an anti-mouse-Cy5 (Jackson Laboratories, 715.175.150) diluted 1:500 and an anti-rabbit Alexa 488 (Molecular Probes) diluted 1:800. After several washes in PBS-Tr, DNA was counterstained with DAPI, and embryos were mounted in Prolong Antifade medium (Molecular Probes). Immunostaining was imaged with LSM 880 with SR Airyscan module (Zeiss) using ×40/1.4 NA objective. Airyscan processing was performed using 2D Zen Black v3.2 (Zeiss) prior to analysis. Image acquisition was done with the following settings: 2000 × 2000 pixels, 16-bit depth.

### Clustering

Raw data from ModENCODE Chip-seq experiments [30] performed on 0–4 h and 14–16 h AEL embryos (Supplementary Table S6) were analyzed to generate BigWig files using deepTools (v3.5.4.post1) bamcoverage package [31] with default parameters, excluding regions (-bl) identified as blacklisted in dm6 [32]. bigwigAverage from deepTools (v3.5.4.post1) package was then used to average duplicates. Then, the bigwigCompare package from deepTools (v3.5.4.post1) was used to obtain the log2 ratio BigWig file between the Chip-seq averaged BigWig and the Input average BigWig. From this, the enrichment signal of the histone modifications H3K4me3, H3K9ac, H3K27ac, H3K4me1, H3K27me3, and H3K9me3 from 0 to 4 h AEL and H3K4me3, H3K9ac, H3K36me3, H3K27ac, H3K4me1, H3K36me1, H3K27me3, H3K9me2, and H3K9me3 from 14 to 16 h AEL were computed on 5 kb windows across the *D. melanogaster* genome (BDGP6.46) using the computeMatrix Package from deepTools (v3.5.4.post1) with scale-regions mode and the the (–averageTypeBins) option with a 50 bp interval. The clustering of two sets of data was performed using the plotHeatMap package from deepTools (v3.5.4.post1) using the option (–kmeans).

### Distribution of LTR-RTEs insertions relative to genomic features and chromatin states

Using the chromosomal gene and exons annotations of *D. melanogaster* genome (BDGP6.46) available on Ensembl Biomart [33] except for the Y chromosome, we partitioned the genome in three mutually exclusive regions corresponding to exons, introns, and intergenic regions. Exons were already annotated in a bed file [33]. Introns were defined as genomic regions that are present in the gene bed file and which are not in the exon bed file. Intergenic regions are defined as genomic regions that do not overlap with the gene bed file. Using this partition and our annotations of LTR-RTEs insertion sites, we then determined the number of copia, roo, gtwin, and ZAM insertions occurring in these three categories of genomic regions (Supplementary Table S5). To determine whether a specific structure (Intergenic, Intron, and Exon) is enriched or depleted for insertions of each considered LTR-RTE, bilateral binomial statistical tests were performed. To do so, the size of each structure relative to the genome was computed using bedtools genomecov (v2.27.1) [34] default parameters, defining the relative size of intergenic regions (pig = 0.314359), introns (pin = 0.418317), and exons (pex = 0.267325) (Supplementary Table S5). Null hypothesis corresponds to the probability for each LTR-RTE family to be inserted in each defined structure due to its proportion in the genome. We used the Benjamini–Hochberg step up procedure to control the FDR, which is defined as the expected value of the proportion of erroneous rejection of the null hypothesis when conducting multiple comparisons.

As for genomic features, the genomic proportion of each identified cluster was computed using bedtools genomecov (v2.27.1) [34] default parameters (Supplementary Table S5). Significant enrichment or depletion of LTR-RTE insertions in the different chromatin states and clusters were calculated using bilateral binomial statistical test considering the null hypothesis as the probability of insertion in a given state for each LTR-RTE family to be equal to the relative size of this state within the genome. As in the previous part, we used the Benjamini–Hochberg step up procedure to control the FDR.

### Analysis of available ChIP-seq datasets for pioneer transcription factors

Raw ChIP-seq data for pioneer transcription factors (Supplementary Table S6) were processed to generate BigWig files using the bamCoverage tool from the deepTools package (v3.5.4.post1), with default parameters and excluding blacklisted regions in the dm6 genome assembly (-bl option) [32]. Replicate signals from ChIP-seq experiments were then averaged using the bigwigAverage tool from the same deepTools package [34] except for Opa for which an averaged BigWig file was already available. The resulting average BigWig files were converted to BedGraph format using the UCSC bigWigToBedGraph [35]. Peak calling was performed using the bdgpeakcal function from MACS2 package (v2.7.1) [36], with the following parameters: (–cutoff) manually defied; (–min-length) 60 and (–max-gap) set to 150. Overlapping regions between the averaged ChIP-seq bed files and the averaged 2–4 h single-cell ATAC-seq bed file were identified using bedtools intersect (v2.27.1) [34]. The genomic coverage of each region was calculated using bedtools genomecov (v2.27.1), as previously described. To assess whether gtwin insertions were significantly enriched or depleted in specific genomic regions, two-tailed binomial tests were performed. Under the null hypothesis, the probability of insertion in a given region was assumed to be proportional to the relative genomic size of that region (Supplementary Table S9).

### Statistical analysis of available sci-ATAC-seq datasets

Normalized BigWig files (Counts Per Million, CPM) from the previously published sci-ATAC-seq atlas [37] were analyzed based on two criteria: (i) the dataset had to correspond to an identified cell type, and (ii) it had to cover at least 70% of the genome. These BigWig files were processed using the computeMatrix tool in reference-point mode from deepTools (v3.5.4.post1) [31] to compute signal distributions centered on the 101 ZAM and 210 gtwin insertion sites, spanning 2 kb upstream and downstream (4 kb total). Regions were filtered and sorted based on their signal intensity scores. Mean read counts across the 4 kb window were calculated using the (–averageTypeBins) option, with a bin size of 50 bp.

To analyze globally chromatin accessibility throughout the 8-time windows of embryonic development, sci-ATAC-seq signals (200 bp window) centered around ZAM or gtwin insertions were averaged for each defined cluster of each time window. The same technique was applied to 100 randomly selected regions of a 200 bp window. Ratios between the average sci-ATAC-seq ZAM (or gtwin) signals and random ones result in a single data per cluster in a defined time window. Data corresponding to the same time window were used to generate boxplots and statistical analysis.

For the precise analysis of chromatin accessibility profiles in ±2 kb window around gtwin and ZAM insertion sites in 0–2 h embryo clusters, random profiles were used as a control. These profiles were generated by creating 100 replicates, each consisting of averaged profiles computed from 100 randomly selected genomic regions (4 kb windows). Signal matrices for gtwin, ZAM and the random profiles were computed using computeMatrix in reference-point mode, as described above. The plotProfile tool with the (–outFileNameData) option was used to extract the average read count distribution across all selected regions. The final signal matrix, representing the mean profile, was then visualized using plotProfile (deepTools v3.5.4.post1).

To determine the number of gtwin insertions shared between the first four clusters found in the 0–2 h window of embryonic development, bed files containing gtwin insertion positions were created for each cluster as described before and visualized with a Venn Diagram.

To quantify the number of gtwin insertions located in open chromatin regions during the 2–4 h embryonic stage, BigWig files corresponding to the different clusters were averaged using the BigWigAverage tool from the deepTools package (v3.5.4.post1). Bed files were then generated from the resulting averaged BigWig file, following the procedure described above.

### Isolation of embryonic cells and cell sorting by flow cytometry

The embryonic cells were isolated as previously described [38]. Briefly, overnight laid embryos from *vas*::EGFP line [21] were collected at 25°C and dechorionated in 2.6% bleach. Dechorionated embryos (i.e 400 mg) were transferred in a 7 ml of Tenbroeck tissue grinder WHEATON^™^ filled with 6 ml of Schneider’s insect medium for homogenization with two slow strokes before a 700 *× g* centrifugation for 10 min at 4°C. The pellet was resuspended in 4 ml of PBS 1× containing 0.1% of Trypsin-EDTA and incubated at room temperature for 20 min. The addition of 4 ml of ice-cold PBS 1× containing 20% fetal bovine serum (FBS) is sufficient to stop Trypsin reaction before a 700 × *g* centrifugation for 10 min at 4°C. Pellet containing separated embryonic cells was resuspended in Schneider’s insect medium (2 ml) and filtered in a 40 μm mesh before the addition of 1 ml of Schneider’s insect medium. A final filtration in a 20 μm mesh was performed before cell sorting by flow cytometry. Embryonic cellular samples were analyzed using a 4-Laser-V16-B14-R8YG10 Aurora spectral cell sorter (Cytek, Biosciences, USA) to sort GFP-positive primordial germ cells (PGCs) from GFP-negative somatic cells through a measurement of complete fluorescence spectrum of individual cells. GFP signal was determined by a 488 nm excitation line and detected in its full spectrum emission with B1 as peak channel (498–518 nm). A total of 2.5 × 10^5^ events were recorded per sample and analyzed using the SpectroFlo software version 1.2.1 (Cytek, Biosciences USA). To define and sort the target cell populations (GFP-positive cells), three successive steps of gating were applied. First, cells were gated using the two physical parameters FSC and SSC excluding dead cells and debris. Second, doublets were excluded by comparing the width versus the area of SSC and FSC. Finally, FSC dot plot and GFP signal reported as percentage in positive or negative cells were used to gate and sort the two populations. Live cell sorting experiments were performed at 4°C with a 70 μm nozzle that allows sorting at high speed (2 × 10^4^ events per second). Sorted cells were collected into PBS containing 20% FBS prior to a final centrifugation at 700 × *g* at 4°C and a −80°C freezing in DMSO supplemented with FBS.

### ATAC-seq experiments and analysis

ATAC-seq experiments were performed using the ATAC-seq kit from Diagenode (catalog no. C01080002). Input material was between 100 000 to 130 000 cryopreserved PGCs (GFP-positive) cells isolated from whole embryos. Tagmentated DNA was amplified by PCR using 13 cycles and the purified DNA libraries were sequenced (paired-end sequencing 150 bp, roughly 2 Gb per sample) by Novogene (https://en.novogene.com/). ATAC-seq were performed in duplicates, following Encode’s standards (https://www.encodeproject.org/atac-seq/#standards). After initial quality checks of the sequencing data from the PGC (GFP-positive cells) and the somatic cells (GFP-negative cells) using FastQC (v0.12.1), the adapters (CTGTCTCTTATACACATCTNNNNNNNN) were trimmed using cutadapt (v4.2). Cleared reads were aligned to the *Drosophila* genome (Dmel_R6.32 release) using bowtie2 (v2.5.1). Duplicate alignments were removed using the fixmate and markdup packages of samtools (v1.17). The read coverage normalized by RPKM (–normalizeUsing) were computed using bamCoverage package from deepTools (v3.5.4.post1) with default parameters, excluding regions (-bl) identified as blacklisted in dm6. Chromatin accessibility averages for the duplicates were calculated using the bigwigAverage tool from deepTools package (v3.5.4.post1) to generate an averaged bedgraph. The chromatin accessibility in a region spanning 2-kb upstream and downstream of ZAM insertions and 100 random genomic positions were computed with the ComputeMatrix package from deepTools (v3.5.4.post1) as described above. The mean signal obtained from the duplicate was then computed using Matlab (R2024). To perform a peak calling, the BigWig files were converted into BedGraph files using USCS tool bigWigToBedGraph [35]. The peak calling was then computed using macs2 (v2.2.7.1) bdgpeakcall package [36] with the following parameters: (–cutoff) manually set; (–min-length) 60 and (–max-gap) 150. The bedtool subtract package [34] was used to identify the genomic regions that are only open in the PGC (GFP-positive) cells using the bed file generated using the sequencing of the somatic cells (GFP-negative). To determine the number of gtwin insertions shared between the first four clusters found in the 0–2 h window of embryonic development and the PGC, bed files were created as described before and visualized with a Venn Diagram (Supplementary Table S8).

### Statistical analyses and visualization

Statistics and data visualization were performed using the ggplot2 (v3.4.3) and eulerr (v7.0.2) libraries (https://ggplot2.tidyverse.org) on R (v4.3.1) (https://www.R-project.org/) and Matlab (R2024).

## Results

### Four LTR-RTEs exhibit transcriptional and insertional activity across successive generations

Our previous qPCR analysis suggested a progressive genomic copy number increase of two LTR-RTEs, ZAM and gtwin, after 30, 41, and 72 successive generations of conditional piwi-sKD (i.e G30, G41, and G72) [20]. For these experiments, genomic DNA was extracted from second filial generation (F2) embryos laid by flies reared at a nonpermissive temperature (20°C), which allows somatic Piwi expression, while their grandparents had been exposed to a permissive (25°C) that impairs Piwi expression (Supplementary Fig. S1) [20]. In the present study, we employed long-read genomic sequencing to provide a comprehensive analysis of LTR-RTE genomic insertions. Genomic DNA was extracted from 100 adult males of F2 progenies from the G11, G31, or G73 populations (Fig. 1A and Supplementary Table S1). Additionally, we sequenced the genome of the original G0 strain, and a G0-derived strain that had been maintained at 20°C for 100 generations (G0F100).

To annotate LTR-RTE genomic insertions, we employed the TrEMOLO method, recently developed by our group [24]. Comparisons of annotated insertions for G11, G31, G73, with the parental G0 strain revealed 280, 514, and 798 novel LTR-RTE insertions, respectively, spanning 43 distinct LTR-RTE families (Fig. 1B and Supplementary Table S2). Notably, the number of genomic insertions of ZAM and gtwin increased significantly and constantly from G11 to G73 (Fig. 1B), which is in line with our previous findings [20]. The number of copia insertions also increased but at a slower rate compared to ZAM and gtwin. Insertions of roo and rover increased between G11 and G31 but remained relatively stable thereafter (Fig. 1B and Supplementary Table S2). Interestingly, the number of insertions for copia, roo, and rover also increased in the G0F100 strain, which was continuously reared at 20°C. This observation suggests that these three families can transpose spontaneously at 20°C in our strain, in the absence of any piwi-sKD treatment applied to the grandparents of the sequenced F2 males (Fig. 1B).

Some of these spontaneous insertions may have occurred during the development of these males, raised at 20°C, possibly in their somatic cells. The case of rover supports this hypothesis, as the frequency of new rover insertions remained consistently very low (Supplementary Table S3), and no evidence of vertical transmission was observed. Indeed, unlike certain copia and roo insertions that were shared between small samples of sequenced genomes and likely represent germline-inherited insertions, no single rover insertion was detected at the same genomic position in at least two samples (Supplementary Table S4). Based on these data, we concluded that ZAM, gtwin, roo, and copia produced new germinal insertions that were vertically transmitted to the analyzed males.

To confirm the induced derepression of ZAM and gtwin and the spontaneous expression of copia and roo under piwi-sKD conditions, we analyzed the impact of piwi-sKD on piRNA levels in fly samples. We hypothesized that under permissive conditions for piwi-sKD, not only would Piwi levels decrease, as previously shown [20], but the levels of antisense piRNAs targeting the induced families (ZAM and gtwin) would also be reduced, while those targeting the noninduced families (copia and roo) would remain unaffected. To test this, we performed small RNA sequencing to compare antisense piRNA levels against these four families in G0F100 ovaries under permissive 25°C and nonpermissive 20°C conditions. We observed that the levels of copia and roo piRNAs were slighty increased after Piwi depletion for unknown reasons. In contrast, the levels of ZAM and gtwin piRNAs were drastically reduced by 5- and 6-fold, respectively (Fig. 1C).

We next conducted single-molecule inexpensive fluorescent *in situ* RNA hybridization (smiRNA-FISH) to examine LTR-RTE expression levels in the ovaries at 20°C and 25°C (Fig. 1D). Interestingly, our analysis revealed that roo and copia transcripts were detected in the ovaries regardless of temperature (Fig. 1D). The roo transcripts accumulated in the oocyte cytoplasm at both temperatures, as it has been previously observed for transcripts of the LINE-RTE I-element [39, 40]. The copia transcripts were detected in the nuclei of the follicle cells. Contrary to roo and copia, ZAM and gtwin are clearly not expressed in the ovaries at 20°C but start to express in the follicle cells of the ovaries at 25°C when Piwi is depleted (Fig. 1D). ZAM expression is restricted to the posterior follicle cells as previously reported [15, 41], whereas gtwin seems to have a broader expression pattern throughout the follicle epithelium (Fig. 1D). Overall, our approach enabled us to accumulate a substantial number of new germline insertions for four actively transposing LTR-RTEs (ZAM, gtwin, roo, and copia) and to observe that each appears to occupy a distinct expression niche within the ovary.

### Distinct chromatin niches for genomic insertions of four LTR-RTEs

We next investigated potential biases in the genomic insertion sites of the four LTR-RTEs. For this analysis, we selected the G73 sequence dataset due to its higher number of insertions compared to other datasets. Since we worked with large populations of flies harboring polymorphic LTR-RTEs insertions, selection could have favored the survival of individuals with beneficial insertions, potentially at the expense of those with neutral or deleterious ones. To test for evidence of positive selection, we first estimated the frequency of each new insertion in the G73 population using our long-read-sequencing data. Our analysis revealed that most insertions were segregating at low frequencies in the population (Supplementary Fig. S2A). Of note, two insertions—one from roo and another from copia—appear to have undergone positive selection, as their frequencies have reached ∼50% in the population. The elevated frequency of these insertions make them strong candidates for further investigation. We then focused on another indication of potential positive selection: the integration of LTR-RTE into piRNA clusters. Indeed, among the new insertions, those occurring in piRNA clusters are of particular interest, as these regions are known to act as sources of piRNAs [26]. Such insertions are expected to influence the piRNA population, favoring the production of piRNAs that silence the expression of the corresponding TE [42]. However, as shown in Supplementary Fig. S2B, none of the 17 new insertions identified in annotated piRNA clusters appeared significantly more frequent than the others (Supplementary Fig. S2C). Altogether, no or few beneficial mutations caused by recent insertions were detected.

**Figure 2.**
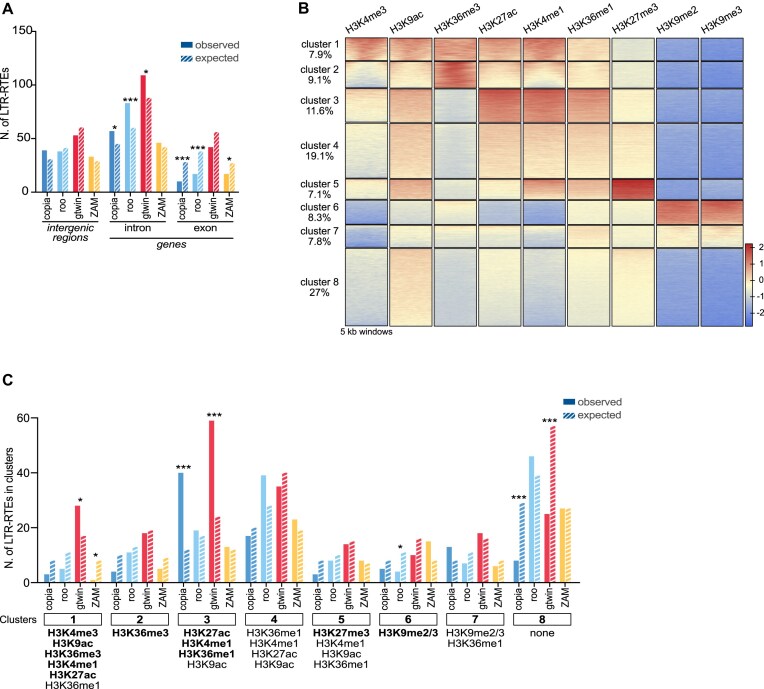
Each LTR-RTE species has its own specific chromatin domain preferences for genomic integration. (**A**) Bar plots showing the observed (filled bars) and expected (dashed bars) numbers of new LTR-RTE insertions in the intergenic, intronic, and exonic regions of the genome. Expected values were calculated based on the proportional size of each genomic region (Supplementary Table S5) and the total number of new insertions identified for each LTR-RTE species. Statistical significance was assessed using binomial tests corrected with the Benjamini–Hochberg procedure to control the FDR; *P*-values: * < 0.05, *** < 0.001. (**B**) Heatmap illustrating genome-wide clustering of nine post-translational histone modifications based on ChIP-seq data from 14 to 16 h *Drosophila* embryos, segmented into nonoverlapping 5 kb genomic bins. This analysis identified eight distinct clusters, each representing a defined proportion of the genome (indicated on the left). The intensity of ChIP-seq signal for each histone modification is displayed using a color gradient (shown at the bottom right) with red indicating an enrichment and blue indicating a depletion. (**C**) Bar plots displaying the observed (filled bars) and expected (dashed bars) numbers of LTR-RTE insertions for each species in each cluster, as defined in panel (B). Expected values were calculated similarly as in panel (A) considering the genomic proportion of each chromatin cluster (Supplementary Table S5). Statistical significances (*P*-values) were calculated using binomial tests corrected by the Benjamini–Hochberg step up procedure to control the FDR; *P*-value: * <0.05, ** <0.005, *** <0.001.

Moreover, purifying selection is also known to be stronger against insertions into genes, and even more into coding sequences, than into intergenic regions [43–46]. To determine the distribution of newly integrated LTR-RTEs, we partitioned the *D. melanogaster* genome into intergenic, intronic, and exonic regions, and quantified the number of LTR-RTE insertions within each category (Fig. 2A). As a reference, “expected” values correspond to the proportional sizes of these three genomic bins (Supplementary Table S5). The number of LTR-RTE insertions in the G73 dataset was then distributed according to these proportions. For example, the “observed” number of intronic insertions for gtwin slightly but significantly exceeded the “expected” values, whereas the “observed” number of exonic insertions was somewhat lower than the “expected” one, although this difference was not statistically significant. These trends were slightly more pronounced for the other three LTR-RTEs, likely because their insertions occured, on average, before those of gtwin and have therefore been subjected to purifying selection for a longer period. However, the degree of exonic depletion observed here was always much lower than what has been reported for much older germline insertions that have undergone extended periods of purifying selection in natural populations [44]. Instead, the observed patterns in our G73 dataset were more reminiscent of those of recent insertions that would not have yet been totally eliminated by purifying selection [17].

Therefore, despite the filtering effects of natural selection, most of the recent insertions remain detectable. Accordingly, we investigated whether specific chromatin landscapes could impact the insertion site choice for each of the four LTR-RTEs. As a first approach, we used available chromatin immunoprecipitation sequencing (ChIP-seq) datasets from 0 to 4 h after-egg-laying (AEL) embryos, provided by ModEncode (Supplementary Table S6), to partition the genome into distinct chromatin clusters based on epigenetic features. This genome-wide partitionning was performed across 5 kb windows, following previously established methods in S2 cells [47]. However, as shown in Supplementary Fig. S3, the epigenetic profiles at this early embryonic stage did not allow a clear separation between active and repressive chromatin domains. These observations are consistent with previous studies showing that chromatin architecture is emerging progressively during embryogenesis, evolving from an initially unstructured state to more defined domains as development progresses [48, 49]. In line with this, using ModEncode ChIP-seq datasets from 14 to 16 h AEL embryos (Supplementary Table S6), we could clearly partition the genome into 8 distinct clusters, each defined by unique epigenetic profiles (Fig. 2B). The improved chromatin resolution observed at 14–16 h AEL provided a robust framework for an accurate analysis of LTR-RTE insertion site preferences. The G73 insertions of the four LTR-RTEs were annotated and distributed in the eight distinct clusters. The significance of their respective distributions was calculated based on the exact number of insertions expected for each LTR-RTE from the proportion of the genome occupied by each of these eight chromatin clusters (Supplementary Table S5).

The four LTR-RTEs were detected across all clusters; however, significant enrichment was observed in clusters 1–4 (Fig. 2C). These clusters are characterized by histone marks commonly associated with open chromatin, including H3K4me1, H3K9ac, and H3K27ac. Notably, each LTR-RTE exhibited a distinct distribution pattern. Gtwin showed pronounced enrichment in clusters 1 and 3, while copia displayed marked enrichment in cluster 3. In contrast, roo and ZAM displayed a weak preference for cluster 4. Overall, this analysis reveals some partitioning of the open chromatin genomic compartment into distinct LTR-RTE-specific niches.

### Gtwin and ZAM exhibit distinct timing of genomic insertion during embryogenesis

**Figure 3.**
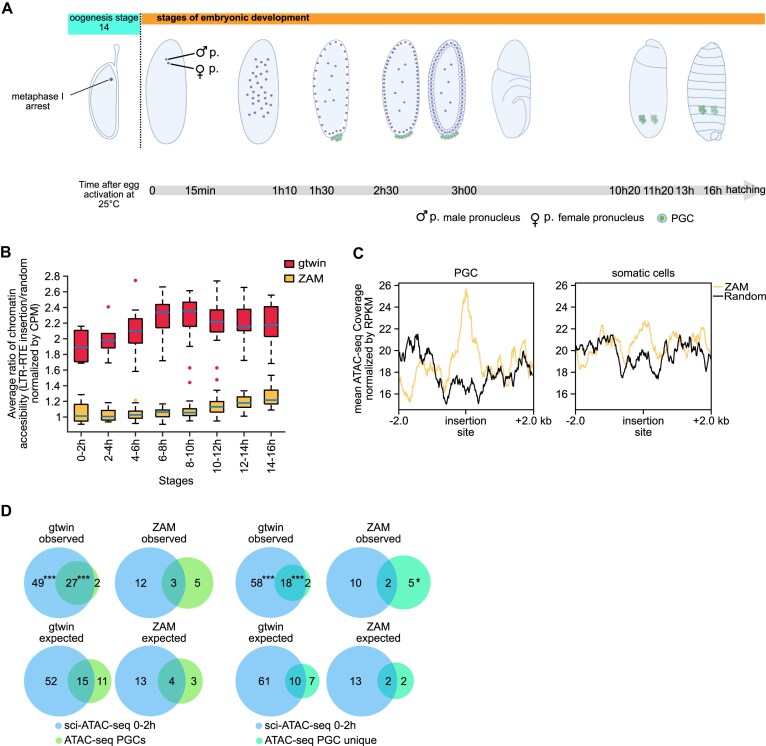
Differential timing of integration during embryogenesis. (**A**) Schematic representation of the last stage of oogenesis containing the arrested oocyte in metaphase I and the different stages of *Drosophila* embryonic development before hatching. Time AEL is indicated at the bottom. PGCs are in green. (**B**) Boxplots representing the temporal kinetics of the average chromatin accessibility around gtwin (red) and ZAM (orange) insertions, relative to random profiles normalized by CPMs uniquely mapping reads. For each time window, the ratio of accessibility is defined as the average sci-ATAC-seq signal of the 200 bp windows centered on LTR-RTE insertions for each defined cluster (at each time window) divided by the average signal obtained for 100 randomly selected 200 bp windows in the same cluster. (**C**) Metaplots showing mean ATAC-seq signals within 4 kb windows centered on 101 *ZAM* insertion sites (yellow) and 100 random insertion sites (black). The signals are averaged from two replicates and normalized by coverage (RPKM: Reads Per Kilobase of transcript per Million mapped reads). The left panel shows data from PGCs, while the right panel displays data from somatic cells, both sorted out of overnight embryos. (**D**) Venn diagrams illustrating the distribution of observed (top) and expected (bottom) gtwin or ZAM insertions in the chromatin accessibility domains detected in 0–2 h embryos (sci-ATAC-seq pooled data, in blue) and in PGCs of late embryos (ATAC-seq, in green). The numbers are those of the insertions that are within stage-specific ATAC-seq peaks as well as those that are located in chromatin domains that are accessible at both stages of embryonic development (overlaps). The right panels (PGC unique) are missing those ATAC-seq PGC peaks that are also present in the somatic cells of the corresponding late embryos. The expected values are based on random distribution, adjusted to the size of each defined regions in the whole genome (Supplementary Table S8). Significant enrichments in the observed insertions are indicated with asterisks. *P*-value: * <0.05, *** <0.001.

To investigate the timing of the LTR-RTE genomic insertions during development, we focused on the sequences of events from oogenesis to embryogenesis. Oocytes undergo meiosis and arrest at metaphase I during the final stages of oogenesis. Upon ovulation, the oocytes are released into the oviduct, where they become activated [50] (Fig. 3A). This activation involves resumption of meiosis, fertilization, and initiation of embryogenesis. Previous studies have shown that gypsy/mdg4, an infectious, enveloped LTR-RTE belonging to the Gypsy clade, is expressed in follicular cells during oogenesis. However, its insertion into the germline genome occurs later, during embryogenesis [51]. This ability of gypsy/mdg4 to transpose in the embryo has been leveraged to design a quantitative assay of gypsy/mdg4 mobilization [52]. Given these prior findings, and the facts that both gtwin and ZAM are enveloped similarly to gypsy/mdg4, we hypothesized that integration of gtwin and ZAM may also occur during embryogenesis. To test this hypothesis, we sought to determine the specific stage of embryonic development during which gtwin and ZAM insertions occur. To this end, we reanalyzed single-cell ATAC-seq (sci-ATAC-seq) data from embryos spanning 0–16 h AEL [37]. We calculated the average chromatin accessibility within a 200 bp window centered on the gtwin or ZAM insertion sites, normalizing these values to signals from 100 randomly selected genomic regions. Our analysis revealed significant enrichment of gtwin insertion sites in open chromatin across all studied time windows, including the 14–16 h AEL window (Fig. 3B), which is consistent with our previous analysis performed on 14–16 h whole embryos (Fig. 2B and C). This analysis further shows that gtwin insertions are also enriched in open chromatin as early as the 0–2 h AEL window (Fig. 3B). For ZAM insertion sites, chromatin accessibility was consistently lower than that of gtwin across all time windows. However, accessibility slowly increased throughout embryogenesis, reaching a 1.1- to 1.2-fold increase during the 10–16 h AEL window (Fig. 3B). At this stage, PGCs are specified (Fig. 3A), suggesting that ZAM integration may occur in PGCs. To investigate this possibility, we performed ATAC-seq experiments on purified PGCs.

Using a *Drosophila* strain expressing GFP-Vasa [21], we separated GFP-positive PGCs and GFP-negative somatic cells from overnight egg collection by fluorescence-activated cell sorting (FACS) [38]. ATAC-seq was performed in duplicate for both cell types. We then analyzed the averaged ATAC-seq signals within a 4 kb window centered on 101 ZAM insertion sites and compared these profiles to 100 randomly selected 4 kb genomic regions in the same cell types. As expected, the averaged ATAC-seq profiles showed a distinct peak centered on the ZAM insertion site, with the signal being more pronounced in PGCs than in somatic cells (Fig. 3C). This observation indicates that ZAM insertion sites are associated with open chromatin domains that are more specific to PGCs than to somatic cells. Taken together, these findings suggest that the accessibility profiles of gtwin and ZAM insertion sites differ during embryonic development.

To confirm our observations, we investigated whether the number of ZAM and gtwin insertions was significantly enriched into open chromatin regions in early embryos (0–2 h AEL) or late PGCs. The expected values were calculated based on the relative size of open chromatin regions identified in the two experiments described above, compared to the whole genome (Supplementary Table S8). Insertions were then classified into three categories: embryo-specific open chromatins, PGC-specific open chromatins, and regions shared by both (Fig. 3D). Gtwin insertions were found to be significantly enriched in embryo-specific and shared open chromatin regions, while the ZAM insertions were predominantly located in PGC-specific open chromatin regions (Fig. 3D, left panels). To refine this analysis, we isolated the PGC-specific chromatin accessibility profile (ATAC-seq PGC unique) by subtracting the ATAC-seq signal of GFP-negative somatic cells from the PGC signal of late embryos (Supplementary Table S8). This revealed that a subset of ZAM insertions were specific to late embryonic PGCs, whereas gtwin insertions were more enriched in the earlier developmental window (Fig. 3D, right panels). These findings indicate that gtwin and ZAM genomic insertions occur at distinct stages of embryogenesis.

### Gtwin preferentially inserts into the open chromatin before cellularization, independently of pioneer factors

**Figure 4.**
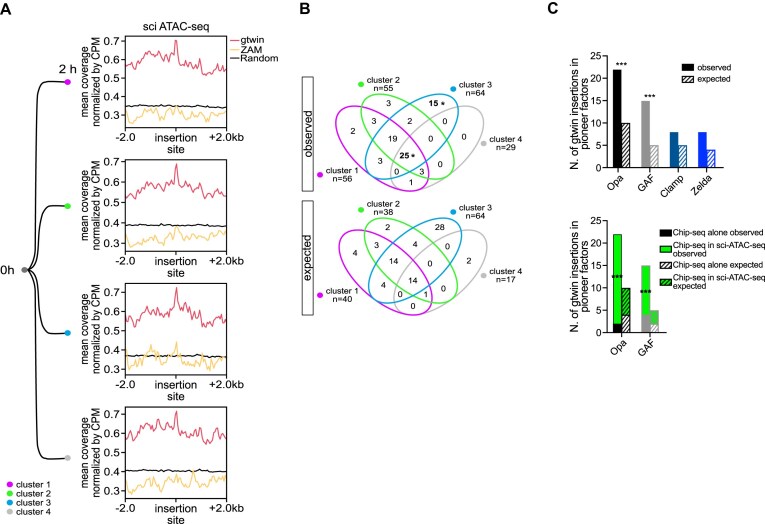
Gtwin preferentially inserts into the open chromatin before cellularization, independently of pioneer factors. (**A**) Metaplots depicting mean coverage of sci ATAC-seq in 4 kb windows centered on 210 gtwin (red), 101 ZAM (yellow), or 100 random insertion sites (black) in the four distinct clusters previously identified in 0–2 h embryos [37]. Values were normalized by CPM unique mapped reads. (**B**) Venn diagram highlighting the distribution of the 76 gtwin insertions sites enriched in sci-ATAC-seq signal among the four clusters. The numbers represent the observed (top) and expected (bottom) count distribution of gtwin insertions in each cluster. The expected values (bottom) are based on random distribution, adjusted for the size of each subset. Significant enrichment or depletion of the insertions observed in two subsets is marked with asterisk. *P*-value: * < 0.05. (**C**) Bar plots showing the observed (filled bars) and expected (dashed bars) numbers of gtwin insertions within pioneer factor binding sites. The upper panel presents the distribution of gtwin insertions across each pioneer factor peaks (Opa, GAF, Clamp, and Zelda ChIP-seq) at 2–4 h AEL. Expected values were calculated based on the genomic coverage of each pioneer factor (Supplementary Table S9). The lower panel shows the observed and expected numbers of gtwin insertions within Opa- or GAF-bound regions, further subdivided according to their overlap or not with open chromatin regions defined by sci-ATAC-seq at 2–4 h AEL (highlighted in green) (Supplementary Table S9). Statistical significance was assessed using binomial tests; ***P < 0.001. (Opa ChIP-seq in sci-ATAC-seq 2–4 h *P*-value 2.404e-6; GAF ChIP-seq in sci-ATAC-seq 2–4 h, *P*-value 1.309e-4).

To better define the timing of gtwin insertion in open chromatin at the very early stage of embryonic development (0–2 h AEL), we decided to analyze chromatin accessibility around gtwin insertions within the four nuclei clusters previously defined by their distinct ATAC-seq profiles [37]. For each cluster, we found that the average chromatin accessibility was enriched within ±2 kb windows centered on gtwin insertion sites, as compared to either random sites or ZAM insertion sites (Fig. 4A). Interestingly, among the 76 gtwin insertion sites found in sci-ATAC-positive regions (Fig. 3D) and analyzed here, 25 of them were shared between the four clusters (instead of 14 expected by chance) (Fig. 4B, upper and lower panel, respectively), suggesting that at least some gtwin insertions might have occurred even before the specification of these four clusters. Altogether our results show that gtwin insertion may occur at the early stage of embryogenesis, before cellularization.

To explore the potential mechanisms driving insertion site selection at this early stage, we next examined whether gtwin insertions are preferentially associated with a pioneer transcription factor. During early embryogenesis, chromatin accessibility is progressively determined by pioneer transcription factors, which bind DNA within nucleosomes and initiate the opening of *cis-*regulatory regions, thereby establishing accessible chromatin domains [53, 54]. We therefore asked whether gtwin insertions are enriched in genomic regions bound by four well-characterized pioneer factors: GAGA factor (GAF), odd-paired (Opa), chromatin-linked adaptor for MSL proteins (CLAMP), and Zelda, using ChIP-seq datasets from 2 to 4 h AEL [55–57] (Supplementary Table S6). This analysis revealed a significant enrichment of gtwin insertions in Opa- and GAF-binding sites (Fig. 4C, upper panel; Supplementary Table S9). However, as shown in Supplementary Table S9 and in the lower panel of Fig. 4C, this enrichment appeared to be restricted to insertions also located within open chromatin regions. This observation was made by partitioning the pioneer factor-associated gtwin insertion sites on the basis of their overlap or not with sci-ATAC-seq peaks from the same developmental window (2–4 h AEL) (Supplementary Table S6). Notably, Opa- and GAF-bound sites that lacked an open chromatin signature were not targeted by gtwin more frequently than by chance (*P* value = 0.4528 and 0.1269, respectively). The preferential targeting of gtwin to GAF- and Opa-bound sites does not therefore appear to result from direct recruitment by these pioneer factors. Instead, it likely reflects a general insertional preference into open chromatin regions, including those rendered accessible by the action of these pioneer factors. Altogether, this suggests that the accessibility of chromatin during early embryogenesis is the main feature contributing to the choice of gtwin insertion sites.

### Late ZAM insertion sites correlate with late ZAM embryonic expression

**Figure 5.**
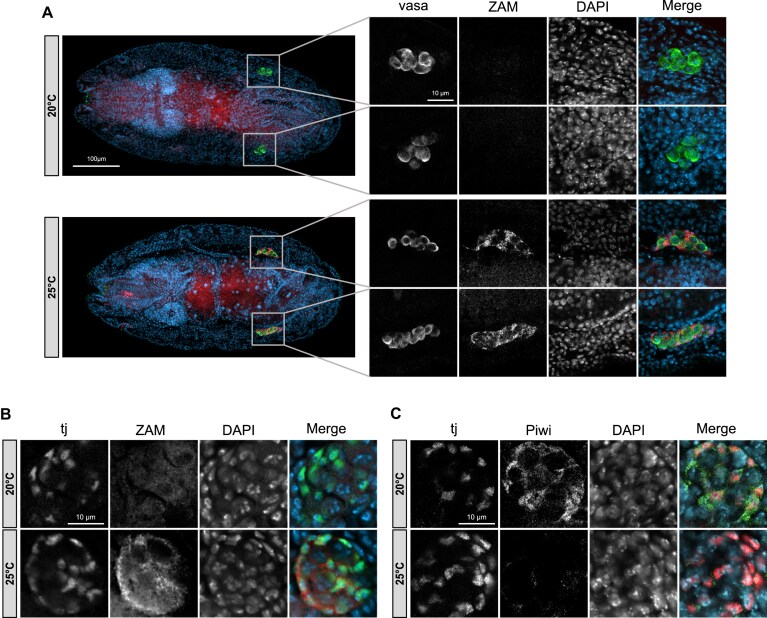
Chromatin accessibility of ZAM insertion sites correlates with ZAM late embryonic expression. (**A**) Vasa immunostaining combined with ZAM smiFISH on 12- to 16-h whole-mount G73 embryos at 20°C (upper panel) and 25°C (lower panel). The right panel shows higher magnification of embryonic gonads. Anti-vasa antibody (green) labeled the PGCs of the gonads. ZAM transcripts labeled in red are detected in cells surrounding vasa-positive cells at 25°C (lower panels). DNA is labeled with 4,6-diamidino-2-phenylindole (DAPI, blue). (**B**) Traffic jam immunostaining combined with ZAM smiFISH of gonadal cells of 12- to 16-h G73 embryos at 20°C (upper panel), 25°C (lower panel). The somatic primordial gonadal cells (SPGs) labeled in green are tj-positive cells. ZAM transcripts labeled in red are detected in tj-positive cells at 25°C (lower panel). DNA is labeled with 4,6-diamidino-2-phenylindole (DAPI; blue). (**C**) Immunostaining of gonadal cells from 12 to 16 h embryos laid by G73 females crossed with males expressing a GFP-tagged version of Piwi, at 20°C (upper panel) or 25°C (lower panel). Traffic jam (red) and GFP (Piwi, green) antibodies revealed the zygotic expression of Piwi in tj-positive cells at 20°C. A decrease in Piwi zygotic expression was observed at 25°C, consistent with the activation of the thermo-inducible RNAi system targeting Piwi.

Two hypotheses could explain the late germline genomic insertion of ZAM. The first hypothesis is that the germ cells infected by ZAM particles during oogenesis remain dormant during early embryogenesis. The second hypothesis postulates the expression of a “second-wave” of ZAM, potentially facilitated by the permissive 25°C temperature at which piwi-sKD is possible. To test this possibility, we combined ZAM smiFISH and germline-specific vasa immunostaining on late embryos. High expression of ZAM was observed in the gonads of embryos laid at 25°C but not at 20°C (Fig. 5A). At these late stages of embryogenesis, the PGCs have migrated away from the midgut toward the adjacent mesoderm and have become associated with somatic gonadal precursors (SGPs) [58, 59] that express traffic jam (tj) [60].

As expected, ZAM expression was specifically detected in the tj*-*positive cells at 25°C (Fig. 5B). Additionally, zygotically expressed Piwi levels were lower at 25°C compared to 20°C, as anticipated (Fig. 5C). Overall, these analyses, showing that ZAM is expressed in tj-positive SGPs cells in embryogenesis and is sensitive to Piwi depletion, strongly support the “second wave hypothesis.” Note however that, although less likely, the alternative hypothesis of maternally inherited dormant ZAM viral particles is not totally ruled out, especially since both hypotheses are not mutually exclusive. Collectively, these results suggest that ZAM insertion sites correlate with a specific chromatin accessibility landscape in late PGCs. We propose that somatic ZAM expression leads to PGC infection, with ZAM invading open genomic regions in late-stage PGCs.

## Discussion

TEs may alternate rapid bursts of activity and prolonged phases of repression during which their replication within the host genome is limited [61]. The interactions observed today between TEs and their host genomes, as well as among TEs themselves, likely reflect the outcomes of extensive co-evolution. This process has enabled the coexistence of several TE families within the same genome while minimizing detrimental impacts on the host. In an attempt to describe the diversity of these interactions, we took advantage of a particular *D. melanogaster* laboratory strain [20] to simultaneously impair the repression of several LTR-RTEs. We observed the efficient germline transposition of four active elements (roo, copia, gtwin, and ZAM). This approach revealed the existence of two distinct categories of LTR-RTEs: ZAM and gtwin, on one hand, whose transposition is induced by the depletion of Piwi in gonadal somatic tissues (Piwi-sKD), and roo and copia, on the other hand, whose activity is independent of this treatment and which transpose spontaneously, even in the presence of Piwi. We found that they all displayed distinct characteristics at various stages of their replication cycles, as follows.

Regarding expression, we observed cell-type-specific patterns for all four elements. For example, roo transcripts were exclusively expressed in the germinal nurse cells, whereas ZAM, gtwin, and copia were transcribed in various somatic follicular epithelial cells. Specifically, ZAM was predominantly expressed at the posterior pole, gtwin was ubiquitously transcribed, and copia transcripts were expressed throughout the follicular epithelium but sequestered into nuclei. Additionally, ZAM displayed a second somatic expression window in the SGPs of late embryonic gonads, emphasizing its distinct temporal and spatial regulation. Concerning the integration step, our approach was based on characterization of the overall epigenetic specificity of their genomic insertion sites. While all four elements predominantly inserted into regions of open chromatin (clusters 1–4), each displayed distinct preferences for specific chromatin clusters associated with different histone modifications. Gtwin showed significant enrichment in clusters 1 and 3, which are associated with the histone modifications H3K4me3 in cluster 1 and H3K27ac and H3K4me1 in cluster 3. Copia was enriched in cluster 3, whereas roo and ZAM exhibited a preference for cluster 4, which is characterized by open chromatin but lacks a distinct enrichment for specific histone modifications (Fig. 2C).

Moreover, our data indicated that the preference for specific genomic insertion sites may follow the differentiation of the chromatin landscape of the cells that are invaded by gtwin and ZAM at different stages of the embryonic development. Indeed, for maternally deposited gtwin, a significant proportion of the insertions seemed to have occurred as soon as their landing sites had begun accessible, at the very beginning of embryogenesis. Conversely, consistent with the late embryonic wave of ZAM somatic expression, several ZAM insertions were located within different open chromatin regions that were accessible only in late embryonic germ cells. However, although the insertion of a LTR-RTE into closed chromatin is generally considered unlikely, it cannot be entirely ruled out. Therefore, we cannot exclude the possibility that some maternally deposited ZAM virus-like particles would have driven integration at early stages into close chromatin landing sites that would open later in the gonadic PGCs. Altogether, our findings disclosed a novel level of LTR-RTE niche partitioning, linking temporal, and spatial features of the integration step of the replication cycle.

### Further evidence for diversity of expression and integration niches

The diversity of ovarian expression patterns reported here (Fig. 1D) has also been observed recently for 16 families of evolutionarily related LTR-RTEs [15]. These different patterns in the onset of the replication cycles of this class, indicate that each LTR-RTE family has evolved specific host–TE interactions, hijacking tissue-specific transcription factors to adapt their proper expression niche to a specific cell type of the ovary. In our study, we identified a novel cell type in which ZAM is also expressed, the SGPs of late embryonic gonads. Future experiments will be necessary to determine whether the transcription factor called Pointed, which drives ZAM transcription in the posterior part of the ovarian follicular cells [62, 63], is also responsible for its expression in the SGPs.

In our study, we also revealed a novel level of LTR-RTE niche partitioning, at the integration step of the replication cycle. It is well-documented that different TE families, belonging to various classes, exhibit diverse target site preferences due to distinct transposition mechanisms. For example, DNA transposons, like the P-element, manage to create new copies by integrating near the replication origins of the *Drosophila* genome [17, 19], whereas retrotransposons Ty1 and Ty3 specifically insert into Pol III promoters of *S. cerevisiae* [16, 64]. Recently, it has been suggested that LTR-RTEs are rather attracted by open chromatin of active genes, whereas LINE elements, such as the I-element, target AT-rich sites, and tend to integrate near telomeres [17]. These TE-specific host affinities have been described to depend on the enzymes driving their integration such as transposases and integrases. We found here that even LTR-RTEs of the same class, despite using the same integration mechanism, preferentially integrate into open chromatin domains harboring distinct chromatin features (Fig. 2C). This finding suggests that each LTR-RTE familiy has evolved specific interactions between its integrase and host co-factors (DNA- and/or chromatin-binding proteins) providing different affinities for specific genetic and epigenetic marks.

Note that, although specific for each LTR-RTE, their preferred epigenetic landscapes share a common feature, open chromatin, a permissive location for subsequent efficient transcription. These similarities might be considered as cases of concerted evolution by sharing general molecular mechanisms of targeting. A famous mechanism of decompacted chromatin targeting operates via the histone H4 tail that can no longer be targeted by HIV when embedded in closed chromatin [65, 66].

Our data also suggest that the specific integration of LTR-RTEs into distinct epigenetically defined domains might, at least partly, result from different integration timings during development. Strikingly, gtwin and ZAM landing site landscapes correlated with chromatin accessibility data sets extracted from early and late stages of embryogenesis, respectively. The hypothesis, assuming replication cycles with different timings of integration, is supported by the second wave of ZAM expression observed later in embryonic gonads. An obvious difference between these two putative cellular integration niches concerns their ability to proliferate. It is indeed worth noting that, unlike early embryonic nuclei that are rapidly cycling, gonadic germ cells are no longer dividing. As it has been suggested for HIV, further experiments will be necessary to know whether gtwin and ZAM integrases have distinct abilities to be imported into nondividing nuclei.

### A complex interplay of positive and negative selection forces, applied to TEs and their hosts, likely lead to niche partitioning

By studying the simultaneous replication of four LTR-RTEs in the *Drosophila* germline, we observed distinct patterns suggesting that these LTR-RTEs occupy different ecological niches within the TE community. Here, we briefly speculate about the putative selective forces that might have led to host–TE and TE–TE coexistence via niche partitioning.

First, we can notice that the four LTR-RTEs families are expressed in gonadal tissues, the only host compartment supporting vertical transmission of the new TE copies. On the contrary, replication in nongonadal tissues is not only useless for the TE replication but could have been counter-selected by the host as a possible cause of diseases like cancer and aging-related decline [67, 68]. A second type of selective pressure might have prevented toxic TE expression [68] in the germline stem cells, the immortal cell lineage of the gonad. That is probably why ZAM and gtwin are expressed in differentiated somatic gonadal cells, while roo, despite being a germline-specific TE, is expressed in nurse cells, which are differentiated germ cells destined to disappear at the end of oogenesis. Third, on one hand, the new TE copies need to be inserted into the germinal genome, but, on the other hand, the resulting DNA damage may be even more deleterious for the germline survival than the toxicity of the expression step. As a possible trade-off, integration is delayed until the DNA damage-tolerant embryonic stage of development [69], followed by larval stages where germ cell division may compensate for previous cell death [70]. Fourth, further research is needed to characterize the putatively detrimental phenotypic effects of the TE insertions we studied and determine if their preferred integration sites correspond to safe havens within the host genome. Similarly, regarding TE–TE interactions, it is unknown whether the TE-specificity of these integration niches results from detrimental fitness effects of competition between different TE families for common insertion sites. Finally, our nonoverlapping TE expression patterns are in agreement with previous observations [15] suggesting that such a competition between somatic TEs might have led to expression niche partitioning. In conclusion, TE niche partitioning highlights the complex interplay of positive and negative selection forces applied to TEs and their hosts leading to their stable coexistence.

## Supplementary Material

gkaf516_Supplemental_File

## Data Availability

Long reads sequencing data previously published and presented in this study have been deposited at ENA (https://www.ebi.ac.uk/ena) under the accession numbers ERP122844 and PRJEB75331, respectively. The source code of TrEMOLO as well as all the accessory codes are available at https://github.com/DrosophilaGenomeEvolution/TrEMOLO and https://doi.org/10.5281/zenodo.15328208. The ATAC-seq and small-RNA-seq raw data are available on GEO under accession number: GSE274394.
