# Supplementary material for: Temporal and spatial niche partitioning in a retrotransposon community of the Drosophila melanogaster genome
Source: Nucleic Acids Res. 2025 Jun 12;53(11):gkaf516. doi: 10.1093/nar/gkaf516 (PMC12159745; doi:10.1093/nar/gkaf516)
Supplement: gkaf516_Supplemental_File [file gkaf516_supplemental_file.pdf]

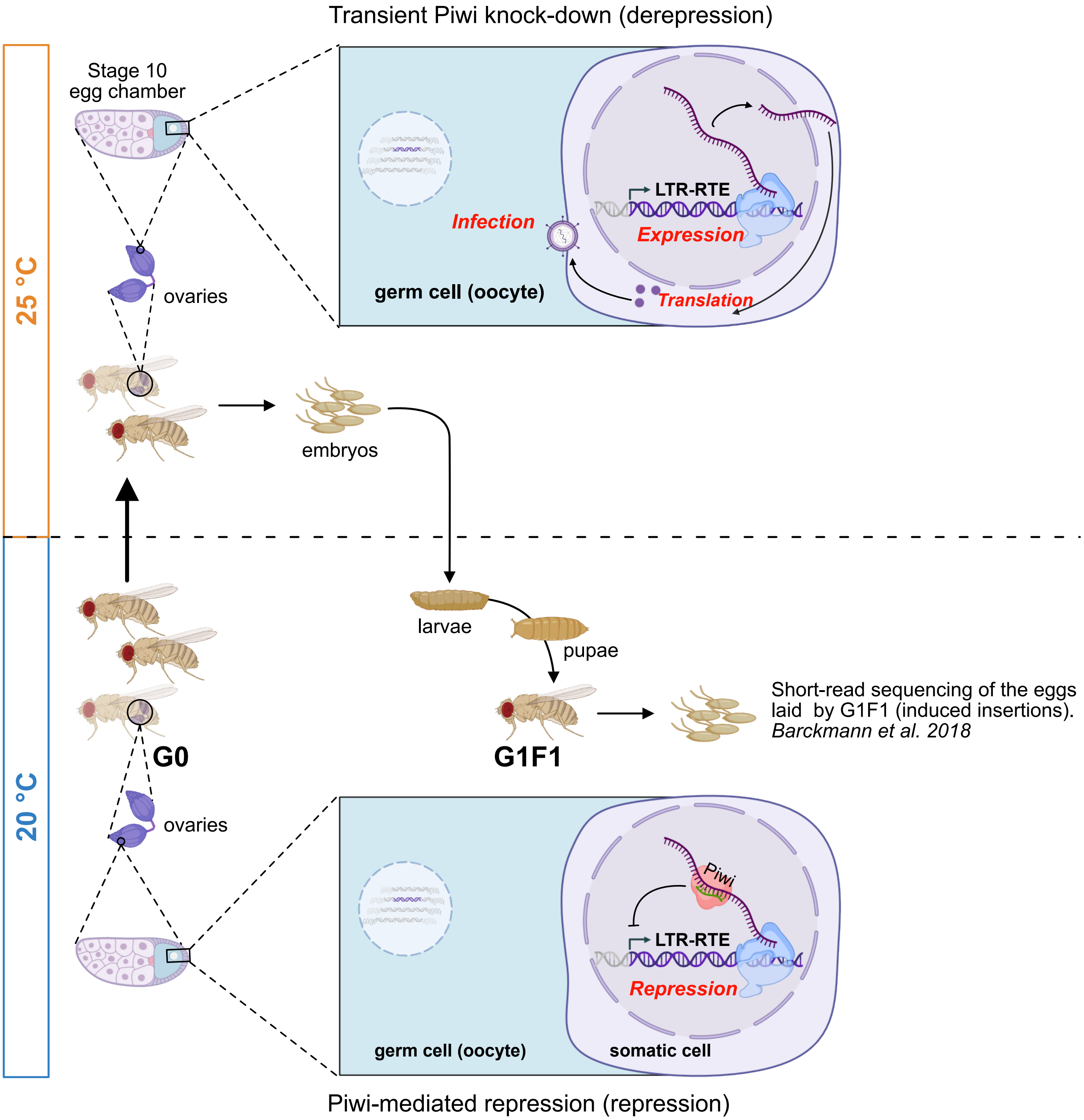

Supplementary Figure S1: Genetic model. Schematic representation of our working model and the previous data published in Barckmann et al, 2018. Lower panel: Piwi-dependent repression of LTR-RTEs at 20°C. Upper panel: Transient Piwi knock-down, by temperature change from 20°C to 25°C applied to adult flies for 5 days, allowing LTR-RTE transcriptional derepression in somatic ovarian cells (purple), completion of the LTR-RTE replication cycle, including the production of viral parti-cles that infect the germline (oocyte, blue). The embryos are then maintained at 20°C for the rest of development. The eggs laid by the G1F1 were sequenced using short-reads to detect newly integrated LTR-RTE in the germline. Created with BioRender. MUGAT, B. (2025) https://BioRender.com/4t1ctqk

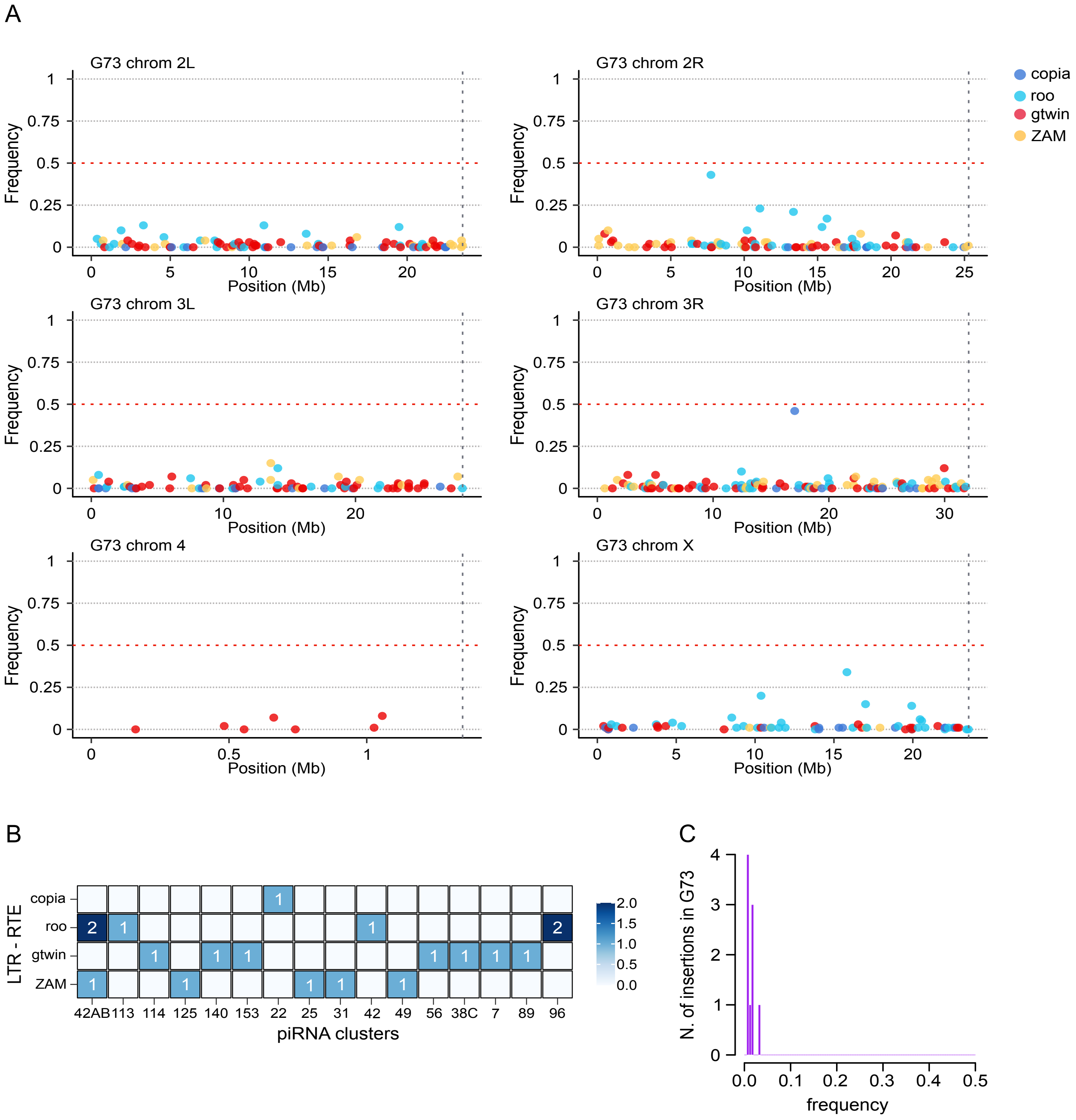

Supplementary Figure S2 : new LTR-RTE insertions are not positively selected in G73 generation : (A) Metaplots depicting the frequency of each new LTR-RTE insertion according to its positioning along each chromosome. Chromosomes 2 and 3 are separated in two metaplots corresponding the left and right arms of these chromosomes. Each family of LTR-RTEs is represented with a color code indicated on the top right-hand side. (B) Table indicating the numbers of LTR-RTE insertions in the different piRNA clusters. (C) Frequency of each LTR-RTE insertion found in piRNA clusters.

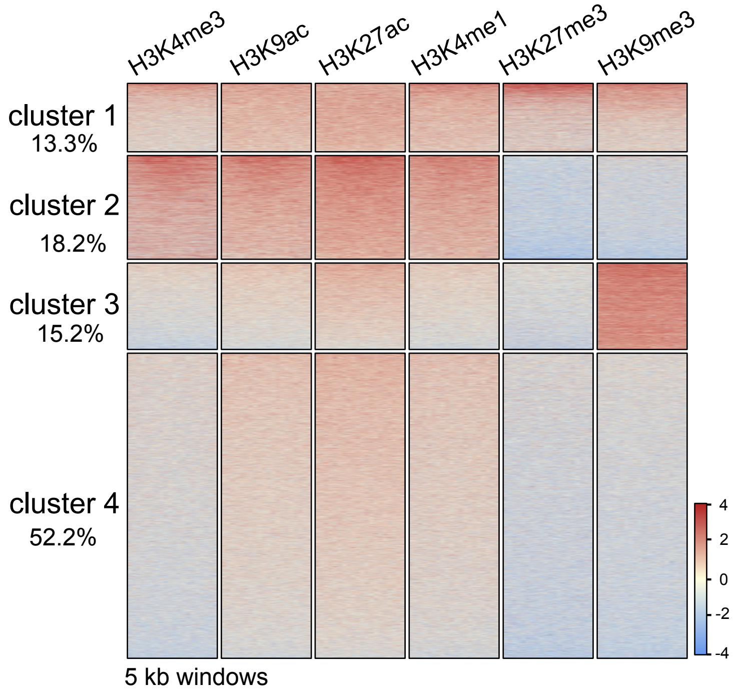

Supplementary Figure S3: Heatmap illustrating genome-wide clustering of six post-translational histone modifications based on ChIP-seq data from 0-4 hrs AEL embryos, segmented into non overlapping 5 kb genomic bins. This analysis identified 4 distinct clusters, each representing a defined proportion of the genome (indicated on the left). The intensity of ChIP-seq signal for each histone modification is displayed using a color gradient (shown at the bottom right) with red indicating an enrichment and blue indicating a depletion.

Name	Numbers of Reads	N50	Mean QuaI, Phred	Total bases	Depth, in X
G0	2,252,087	3.768	9.1	5,418,397,754	30.1
G0F100	3,358,451	11.5	12.5	16,964,518,776	94.2
G11	1,557,842	16.339	12,34	10,749,554,082	59.7
G31	5,681,195	12.0	12.20	23,420,484,737	130.1
G73	3,567,671	19.65	12.4	24,980,845,478	138.8

All lengths are expressed in bases. Quality is expressed in standard Phred scale.

Supplementary Table S1: Sequencing data statistics

Families	G11	G31	G73
1731	1	1	0
17.6	3	13	13
297	4	11	9
3S18	2	3	3
412	3	15	10
accord	0	2	2
blood	11	18	13
Burdock	0	2	3
Circe	1	0	2
**copia**	**40**	**61**	**107**
diver	1	1	1
Dm88	0	2	1
flea	8	14	14
GATE	1	5	3
**gtwin**	**6**	**19**	**210**
gypsy	1	4	6
gypsy4	1	1	0
gypsy6	1	0	2
gypsy7	1	0	0
HMS-Beagle	0	7	2
HMS-Beagle2	1	0	1
Idefix	3	7	18
invader1	6	8	12
invader2	1	1	0
invader3	0	2	2
invader4	3	4	2
invader6	3	2	7
Max-element	8	4	5
McClintock	1	0	0
mdg1	5	7	8
mdg3	7	8	19
micropia	1	2	2
opus	1	13	8
Quasimodo	3	5	4
**roo**	**104**	**153**	**144**
**rover**	**23**	**48**	**39**
springer	0	0	1
Stalker	4	4	5
Stalker2	1	0	1
Stalker4	9	14	10
Tabor	1	1	1
Transpac	4	8	7
**ZAM**	**6**	**44**	**101**

Supplementary Table S2: Annotation of newly integrated LTR-RTEs in the different generations compared to G0 parental genome.

	rover			roo			copia		
Frequency Range (%)	G11	G31	G73	G11	G31	G73	G11	G31	G73
[0-1]	**2**	**36**	**34**	**2**	**45**	**10**	**1**	**35**	**70**
[1-2]	**20**	**9**	**5**	**41**	**42**	**57**	**17**	**7**	**13**
[2-3]	**1**	**1**	0	**15**	**17**	**26**	**16**	**3**	**10**
[3-4]	0	0	0	**10**	**12**	**11**	**2**	**4**	**6**
[4-5]	0	0	0	**7**	**6**	**9**	**1**	**2**	**3**
[5-6]	0	0	0	**5**	**3**	**5**	0	**2**	**1**
[6-7]	0	0	0	**4**	**4**	**6**	0	**2**	**1**
[7-8]	0	0	0	**2**	**2**	**1**	**2**	**2**	**1**
[8-9]	0	0	0	**6**	**2**	**2**	0	0	0
[9-10]	0	0	0	**2**	**2**	0	**1**	**2**	**1**
[10-20]	0	**1**	0	**9**	**13**	**12**	0	**1**	0
[20-30]	0	0	0	**1**	**3**	**3**	0	0	0
[30-40]	0	0	0	0	**2**	**2**	0	**1**	**1**
[40-50]	0	0	0	0	0	0	0	0	0
[50-60]	0	**1**	0	0	0	0	0	0	0
[60-70]	0	0	0	0	0	0	0	0	0
[70-80]	0	0	0	0	0	0	0	0	0
[80-90]	0	0	0	0	0	0	0	0	0
[90-100]	0	0	0	0	0	0	0	0	0

Supplementary Table S3: Distribution of the rover, roo and copia new insertions according to their sequencing frequencies in the G11, G31 and G73 populations

	copia			roo			rover		
Generation	G11	G31	G73	G11	G31	G73	G11	G31	G73
Total insertion number	40	61	107	104	153	144	23	48	39
Shared Insertion number *	1	1	2	11	7	13	0	0	0

* Shared insertions (that were detected in at least two different generations) were very likely vertically transmitted.

Supplementary Table S4 : Evidence for the existence of copia and roo germinal insertions.

Regions	Proportion of the genome	copia (n = 107)			roo (n = 144)			gtwin (n = 210)			ZAM (n = 101)		
		Obs	Exp	P.adj	Obs	Exp	P.adj	Obs	Exp	P.adj	Obs	Exp	P.adj
Intergenic	0.314359	40	34	0.281411	44	45	0.857967	59	66	0.400694	38	32	0.281411
Intron	0.418317	57	45	0.044382	83	60	7.18924E-4	109	88	0.012066	46	42	0.524310
Exon	0.267325	10	29	1.09180E-4	17	38	1.09180E-4	42	56	0.049317	17	27	0.048480
Clustering 14-16h AEL													
Cluster 1	0.078988	3	8	0.139088	5	11	0.139088	28	17	0.030257	1	8	0.030257
Cluster 2	0.116372	40	12	1.81247E-10	19	17	0.719466	59	24	1.32809E-09	13	12	0.816913
Cluster 3	0.070921	3	8	0.204977	8	10	0.816913	14	15	1	8	7	0.826181
Cluster 4	0.091350	4	10	0.152861	11	13	0.816913	18	19	0.964852	5	9	0.357746
Cluster 5	0.083450	5	9	0.389563	4	12	0.046022	10	17	0.152861	15	8	0.100669
Cluster 6	0.191103	17	20	0.670884	39	28	0.076920	35	40	0.670884	23	19	0.631236
Cluster 7	0.077645	13	8	0.389563	7	11	0.670884	18	16	0.862047	6	8	0.862047
Cluster 8	0.270241	8	29	4.44044E-06	46	39	0.378466	25	57	1.45777E-06	27	27	1

Supplementary Table S5: Distribution of the copia, roo, gtwin and ZAM new insertions according to the genomic or chromatin features of their landing sites. “Obs” and “Exp” refer to the observed and expected numbers of LTR-RTE insertions for each genomic and chromatin features. Adjusted p-values "P.adj" were calculated using binomial tests corrected by Benjamini–Hochberg step up procedure to control the false discovery rate.

Antigen	Type of Data	Tissue	GSE	Reference
H3K4me3	Chip-seq of Histone Modification	Embryo 14-16h	GSE47285	DOI: 10.1126/science.1198374
H3K9ac	Chip-seq of Histone Modification	Embryo 14-16h	GSE55557	DOI: 10.1126/science.1198374
H3K36me3	Chip-seq of Histone Modification	Embryo 14-16h	GSE47256	DOI: 10.1126/science.1198374
H3K27ac	Chip-seq of Histone Modification	Embryo 14-16h	GSE47237	DOI: 10.1126/science.1198374
H3K4me1	Chip-seq of Histone Modification	Embryo 14-16h	GSE47281	DOI: 10.1126/science.1198374
H3K36me1	Chip-seq of Histone Modification	Embryo 14-16h	GSE47241	DOI: 10.1126/science.1198374
H3K27me3	Chip-seq of Histone Modification	Embryo 14-16h	GSE47230	DOI: 10.1126/science.1198374
H3K9me2	Chip-seq of Histone Modification	Embryo 14-16h	GSE47247	DOI: 10.1126/science.1198374
H3K9me3	Chip-seq of Histone Modification	Embryo 14-16h	GSE47246	DOI: 10.1126/science.1198374
H3K4me3	Chip-seq of Histone Modification	Embryo 0-4h	GSE16013	DOI: 10.1126/science.1198374
H3K9ac	Chip-seq of Histone Modification	Embryo 0-4h	GSE16013	DOI: 10.1126/science.1198374
H3K27ac	Chip-seq of Histone Modification	Embryo 0-4h	GSE16013	DOI: 10.1126/science.1198374
H3K4me1	Chip-seq of Histone Modification	Embryo 0-4h	GSE16013	DOI: 10.1126/science.1198374
H3K27me3	Chip-seq of Histone Modification	Embryo 0-4h	GSE16013	DOI: 10.1126/science.1198374
H3K9me3	Chip-seq of Histone Modification	Embryo 0-4h	GSE16013	DOI: 10.1126/science.1198374
**GAF**	**Chip-seq of Transcription factor**	**Embryo 2-4h**	**GSM6045770 GSM6045771 GSM6045772**	**DOI: 10.1038/s41467-023-41408-1**

**OPA**	**Chip-seq of Transcription factor**	**Embryo 4h**	**GSE140722**	**DOI: 10.7554/eLife.59610**
Clamp	Chip-seq of Transcription factor	Embryo 2-4h	GSM4618642<br>GSM4618660<br>GSM4618678	DOI: 10.7554/eLife.69937
Zelda	Chip-seq of Transcription factor	Embryo 2-4h	GSM4618643<br>GSM4618661<br>GSM4618679	DOI: 10.7554/eLife.69937

In bold : transcription factor profiles in bigWig format on the dm6 directly available.

REFERENCE TABLE web link

Structure	Original genome built	Reference	Link
Genes	dm6	https://doi.org/10.1093/nar/gkac958	https://www.ensembl.org/biomart/<br>/836e762aea9a306041c6e5ed15cd59e7
Exon	dm6	https://doi.org/10.1093/nar/gkac958	https://www.ensembl.org/biomart/martview/836e762aea9a306041c6e5ed15cd59e7
Chromatin accessibility single cell ATAC-seq	dm6	DOI: 10.1126/science.abn5800	https://shendure-web.gs.washington.edu/content/members/DEAP_website/public/ATAC/revision/bigwigs/
Blacklist of the dm6	dm6	DOI: 10.1038/s41598-019-45839-z	https://github.com/Boyle-Lab/Blacklist/raw/master/lists/dm6-blacklist.v2.bed.gz

Supplementary Table S6 : Key Resources Table

smiFISH-primers	sequences
copia-1-FLAPX	TCATTTAGGTCAGTCATCCTAAACTTTTCCATCCTCCTAAGTTTCGAGCTGGACTCAGTG
copia-2-FLAPX	CTAGCCGCTTGCTTGAGTCCGTAAATTGCCCTCCTAAGTTTCGAGCTGGACTCAGTG
copia-3-FLAPX	TGATCCCTTACTTTCATTCAGGTGATCATCCCTCCTAAGTTTCGAGCTGGACTCAGTG
copia-4-FLAPX	GTAGTCGGACAATACCACGCTTAGTGGCACCTCCTAAGTTTCGAGCTGGACTCAGTG
copia-5-FLAPX	AATTTTATCCATCTCTTCTATTTTTGCACCAGCCTCCTAAGTTTCGAGCTGGACTCAGTG
copia-6-FLAPX	CAAACGGCTTAATATTACGTTTAGCCTTGTCCCCTCCTAAGTTTCGAGCTGGACTCAGTG
copia-7-FLAPX	AGTAACTATCAAATGTGGGTGGTGTGCATTCTCCTCCTAAGTTTCGAGCTGGACTCAGTG
copia-8-FLAPX	GAAGTGTTAACTGATCCAGCATTTGCTGCGCCTCCTAAGTTTCGAGCTGGACTCAGTG
copia-9-FLAPX	AATTTCATTCAGCATTCGATTGGTCGTCTTGCCCTCCTAAGTTTCGAGCTGGACTCAGTG
copia-10-FLAPX	AAGACAAATCACATTATTCTGAACTTGCTCTCCCTCCTAAGTTTCGAGCTGGACTCAGTG
copia-11-FLAPX	TTAGCTCGTTTATGACATGAGGGGTTGTTTGCCCTCCTAAGTTTCGAGCTGGACTCAGTG
copia-12-FLAPX	CAAATAGGGCCATATACTCAGCTTCAGTTGACCTCCTAAGTTTCGAGCTGGACTCAGTG
copia-13-FLAPX	AGGCTGCTACTGAGTTCTGTCTCTTTGTACCTCCTAAGTTTCGAGCTGGACTCAGTG
copia-14-FLAPX	TTGTACTTTTTCTATCAATTTCACTACCAGCCCCTCCTAAGTTTCGAGCTGGACTCAGTG
copia-15-FLAPX	ATTCGGAGTTATTTTTGCTACTATATCTGCTCCCTCCTAAGTTTCGAGCTGGACTCAGTG
copia-16-FLAPX	TGGCTTAAATAGATTTTATCTTCCTGCATCTCCCTCCTAAGTTTCGAGCTGGACTCAGTG
copia-17-FLAPX	AAGTTATTCATTCTTGTCATATCTCCTGTAGCCCTCCTAAGTTTCGAGCTGGACTCAGTG
copia-18-FLAPX	GCGATCAACTGAAGAGTTTACAAACTCACACTCCTCCTAAGTTTCGAGCTGGACTCAGTG
copia-19-FLAPX	TCCTCTTTTAACGTGCCATTTAAGAAAGCTGTCCTCCTAAGTTTCGAGCTGGACTCAGTG
copia-20-FLAPX	TTGGTATTTTTGAGTGAATCCTCGTGCAACCACCTCCTAAGTTTCGAGCTGGACTCAGTG
copia-21-FLAPX	TATGAGCATTTAACTCTGTATTGATGGCTTCTCCTCCTAAGTTTCGAGCTGGACTCAGTG
copia-22-FLAPX	TATTATCCTCTTCATTATAGGATATCTGAGGCCCTCCTAAGTTTCGAGCTGGACTCAGTG
copia-23-FLAPX	CTCTCACTTCTTCTATTAATAATTTCTATGCCCCTCCTAAGTTTCGAGCTGGACTCAGTG
copia-24-FLAPX	ATCAATTCCAATTTCTTTTAAGTGCTCTGCTGCCTCCTAAGTTTCGAGCTGGACTCAGTG
copia-25-FLAPX	TCACTTTCCCTACTCTCATTCGGGTTGCCTCCTAAGTTTCGAGCTGGACTCAGTG
copia-26-FLAPX	ATCTTTCAGGAATTGTATGTTGTCGCATTCCTCCTCCTAAGTTTCGAGCTGGACTCAGTG
copia-27-FLAPX	ATCTTTCAGGAATTGTATGTTGTCGCATTCCTCCTCCTAAGTTTCGAGCTGGACTCAGTG
copia-28-FLAPX	TTTCACTTTCCTTACTATCTTTCAGGAACACTCCTCCTAAGTTTCGAGCTGGACTCAGTG
copia-29-FLAPX	TAATATGCACATAAACAGTTGCACCAAACACTCCTCCTAAGTTTCGAGCTGGACTCAGTG
copia-30-FLAPX	GTTTTAAGTATGGCTTCTTATTGTGCCACATCCCTCCTAAGTTTCGAGCTGGACTCAGTG
copia-31-FLAPX	AAGCTTTTATCTAGCTTTGCACCACTAACCCCTCCTAAGTTTCGAGCTGGACTCAGTG
copia-32-FLAPX	AGCTTTTTCCGTAATGGTTCTTATCATTCTCTCCTCCTAAGTTTCGAGCTGGACTCAGTG
copia-33-FLAPX	GGAAGTCTTGCCTGTTTACCATTTAAACAGGGCCTCCTAAGTTTCGAGCTGGACTCAGTG
copia-34-FLAPX	ATTTCTAATAATTTGCCATCGCTTATATGGCCCCTCCTAAGTTTCGAGCTGGACTCAGTG
copia-35-FLAPX	AATGGTTACACCGCTTTTGTCAAATTCGATCGCCTCCTAAGTTTCGAGCTGGACTCAGTG
copia-36-FLAPX	ACATCCTCCAGTGTAATCTCATGGTCATTCCTCCTAAGTTTCGAGCTGGACTCAGTG
copia-37-FLAPX	AATAAATTCGCCTTGCTTGGCCACTGCCCTCCTAAGTTTCGAGCTGGACTCAGTG
copia-38-FLAPX	GAAACAATCTTTTTTAATGTGGCCTTCTCTGCCCTCCTAAGTTTCGAGCTGGACTCAGTG
copia-39-FLAPX	AGTGTTATTATTGTTGTGCACGATCGCGTTCACCTCCTAAGTTTCGAGCTGGACTCAGTG
copia-40-FLAPX	ATGGCTTAATAGTGACATCTCACTCGATAGCCCTCCTAAGTTTCGAGCTGGACTCAGTG

copia-41-FLAPX	CAGAGAAAGCAAACGTTTTCGCAGCGCCAGCCTCCTAAGTTTCGAGCTGGACTCAGTG
copia-42-FLAPX	GCGACGCCAAACTTTTTCGTTCATAAACGGCCCTCCTAAGTTTCGAGCTGGACTCAGTG
copia-43-FLAPX	ACGAGTCGCTTAGGTACTCTATTATTGTACTTCCTCCTAAGTTTCGAGCTGGACTCAGTG
copia-44-FLAPX	TGCACAACGCTCTGCCTTTTTCCAGGCCTCCTAAGTTTCGAGCTGGACTCAGTG
copia-45-FLAPX	ATTTAATTGTTTATTAGGCATGGACTGGGCCCCCTCCTAAGTTTCGAGCTGGACTCAGTG
copia-46-FLAPX	TTTTAAGTTATTTCAACTGCAACACCAGCACCCCTCCTAAGTTTCGAGCTGGACTCAGTG
copia-47-FLAPX	TTGTAGGTTGAATAGTATATTCCAACACGCCCCCTCCTAAGTTTCGAGCTGGACTCAGTG
copia-48-FLAPX	GATACGGGGAAAACCCAGAAAAACCCGATCACCTCCTAAGTTTCGAGCTGGACTCAGTG
roo-1-FLAPY	TTTTGGATAAGTCTCCACCTATCCAAATTTCCTTACACTCGGACCTCGTCGACATGCATT
roo-2-FLAPY	CTGTTTTATAGATGCAGTTCGCCGCTTTATCTTACACTCGGACCTCGTCGACATGCATT
roo-3-FLAPY	TCTTCATTTCGAATTGGCACCGGAATAAGCCTTACACTCGGACCTCGTCGACATGCATT
roo-4-FLAPY	GTTAACAGTGTATACACCTCCTTAAGTTCCGTTTACACTCGGACCTCGTCGACATGCATT
roo-5-FLAPY	CTGACCGTTTTCCTGGAATTACTAGGTTCTCTTACACTCGGACCTCGTCGACATGCATT
roo-6-FLAPY	TATAATGCCTGCTTGAATTTTTTCGCCTTCTTTACACTCGGACCTCGTCGACATGCATT
roo-7-FLAPY	TCTTCATATTTTCTTCTAATTGCTCTCTATCATTACACTCGGACCTCGTCGACATGCATT
roo-8-FLAPY	TCTTTTTGGCTCTTGGTCAGCCTCATTTTTTTACACTCGGACCTCGTCGACATGCATT
roo-9-FLAPY	CTATTTGCCCTTCTTCTATATTAGGAATTTCCTTACACTCGGACCTCGTCGACATGCATT
roo-10-FLAPY	TTCCATTTATTCCTTTGCTGGAGCGTATGCTTACACTCGGACCTCGTCGACATGCATT
roo-11-FLAPY	AATGGTATTTCATTGACTTAACTCCAGCTTCCTTACACTCGGACCTCGTCGACATGCATT
roo-12-FLAPY	AATAACTCTTGATCTAATTTTCTTGCAGCTCCTTACACTCGGACCTCGTCGACATGCATT
roo-13-FLAPY	TTGATCTTACTTTGACCACTGTTAATCCATGCTTACACTCGGACCTCGTCGACATGCATT
roo-14-FLAPY	ACACAGCTCGAGTTTGGGAATTGTCTTCCTTTACACTCGGACCTCGTCGACATGCATT
roo-15-FLAPY	AGCATATGCTTTTTCGGAGGCGTCCGCTTACACTCGGACCTCGTCGACATGCATT
roo-16-FLAPY	ATCCATTTCCTTAAGTTGAATCCAACTTTCTGTTACACTCGGACCTCGTCGACATGCATT
roo-17-FLAPY	TAATTTATTAGCTTCTTCTACCGAATCAGCTCTTACACTCGGACCTCGTCGACATGCATT
roo-18-FLAPY	GTAAGGCTTGAATCTCTAATCACTGCCTGTTACACTCGGACCTCGTCGACATGCATT
roo-19-FLAPY	TTTTTCTCCAGATTCATGTAACGAGCTATCGCTTACACTCGGACCTCGTCGACATGCATT
roo-20-FLAPY	GTTTCTTTGAATCTCCTAAGGTGACATCCTCTTACACTCGGACCTCGTCGACATGCATT
roo-21-FLAPY	GTGGCTTCATAATTTTCTCCAGAGCCGAGTTACACTCGGACCTCGTCGACATGCATT
roo-22-FLAPY	GTAAATGAGTAACCACATTTCTGGCTTCTCCTTACACTCGGACCTCGTCGACATGCATT
roo-23-FLAPY	GTTCGCTAATTTCTTCTTCGAATAGCGAACTCTTACACTCGGACCTCGTCGACATGCATT
roo-24-FLAPY	TTGCTGCTTCTGCTGCTGCTGTTGTTTTACACTCGGACCTCGTCGACATGCATT
roo-25-FLAPY	CTTCTGCTGCTGGTAGAGGCTCCTTTTTACACTCGGACCTCGTCGACATGCATT
roo-26-FLAPY	CACATCTGCCTATCTTGAGCGGCGAGTTACACTCGGACCTCGTCGACATGCATT
roo-27-FLAPY	CCTTATCTGTGGTCTCCCACTAAGGGATTACACTCGGACCTCGTCGACATGCATT
roo-28-FLAPY	ATATATTCGTGTTCATGTGTGAACATTCTGCCTTACACTCGGACCTCGTCGACATGCATT
roo-29-FLAPY	ACTAAGATTTCAATGGGCCTAGTTTTTCTGGCTTACACTCGGACCTCGTCGACATGCATT
roo-30-FLAPY	AGGTTATTGCTTGCATTCTTTGTTGCACAGCTTACACTCGGACCTCGTCGACATGCATT
roo-31-FLAPY	ACTCCACTAACTTCTCCTATATAAGGTGTTGTTACACTCGGACCTCGTCGACATGCATT
roo-32-FLAPY	ATTCTTCACTTTCGGACTGAATGTTATGGTGGTTACACTCGGACCTCGTCGACATGCATT
roo-33-FLAPY	ACTAGAATTTTATCGTCGGTTCTTGCCGTACATTACACTCGGACCTCGTCGACATGCATT
roo-34-FLAPY	ATGGCGCAATGCTTAAAACTCCTTGCTGTTACACTCGGACCTCGTCGACATGCATT

roo-35-FLAPY	GCTTTAGAGGCTGAATCTCACATGCATTATTTACACTCGGACCTCGTCGACATGCATT
roo-36-FLAPY	TTCGCTGAATTCCAGGGCCAACTTCCTTACACTCGGACCTCGTCGACATGCATT
roo-37-FLAPY	GCATATTCTCTTATTTAGTTGCCATTTCTGACTTACACTCGGACCTCGTCGACATGCATT
roo-38-FLAPY	AATGTGGCTTCCGTCATTATGTGATAGGAATCTTACACTCGGACCTCGTCGACATGCATT
roo-39-FLAPY	ATATTCGGACGTTGTATGCACCATTATTATCCTTACACTCGGACCTCGTCGACATGCATT
roo-40-FLAPY	TGGCAATTTCTTTTTTAAGCTGATTTGGCCTGTTACACTCGGACCTCGTCGACATGCATT
roo-41-FLAPY	ACTTCTGTCATGTTCTCAATTCTTATTTGCATTTACACTCGGACCTCGTCGACATGCATT
roo-42-FLAPY	ACAAGGAACCCATAAATTCGAAAGGAGCACGCTTACACTCGGACCTCGTCGACATGCATT
roo-43-FLAPY	CTCCTAGCGGGTCTAGATATATTGCTGAGTTACACTCGGACCTCGTCGACATGCATT
roo-44-FLAPY	ATATCTTGGTGCTATATCTTTAGGTAATGCGCTTACACTCGGACCTCGTCGACATGCATT
roo-45-FLAPY	CTTGGACATTCCCGATGTCGCTCTAGTTTTACACTCGGACCTCGTCGACATGCATT
roo-46-FLAPY	TTAGTAATGGGTCTAGTGATATATCCTTCCTGTTACACTCGGACCTCGTCGACATGCATT
roo-47-FLAPY	TCTTTACTTTAGCCACTCGGACCTTATCATTACACTCGGACCTCGTCGACATGCATT
roo-48-FLAPY	CTAAAGGCCATCTTGCAGGATGACAATTCTTTACACTCGGACCTCGTCGACATGCATT
gtwin1-FLAPX	TTTATGTCTCTATATGCCGTCCTCCTAAGTTTCGAGCTGGACTCAGTG
gtwin2-FLAPX	GACAAAAACCTACGGGCTCTCCTCCTAAGTTTCGAGCTGGACTCAGTG
gtwin3-FLAPX	CTGCAGTGCTATTTGTAGTACCTCCTAAGTTTCGAGCTGGACTCAGTG
gtwin4-FLAPX	CTGCAGTGCTATTTGTAGTACCTCCTAAGTTTCGAGCTGGACTCAGTG
gtwin5-FLAPX	CAGTCAAAGCCTGGTTAACGCCTCCTAAGTTTCGAGCTGGACTCAGTG
gtwin5-FLAPX	CTCTTAAGTCTGTTTTCCTGCCTCCTAAGTTTCGAGCTGGACTCAGTG
gtwin6-FLAPX	CATCTAATTGCGCCTGGAAACCTCCTAAGTTTCGAGCTGGACTCAGTG
gtwin7-FLAPX	AGTGTTGCATCTGTTGTTTTCCTCCTAAGTTTCGAGCTGGACTCAGTG
gtwin8-FLAPX	TACTTGTGGTGCTTCCACACCCTCCTAAGTTTCGAGCTGGACTCAGTG
gtwin9-FLAPX	AATGTCAAGCTTGATGTCGCCCTCCTAAGTTTCGAGCTGGACTCAGTG
gtwin10-FLAPX	AATGTCAAGCTTGATGTCGCCCTCCTAAGTTTCGAGCTGGACTCAGTG
gtwin11-FLAPX	ATGACACATAGTCATCCTGCCCTCCTAAGTTTCGAGCTGGACTCAGTG
gtwin12-FLAPX	AAGATCTCGTATGCGTCCACCCTCCTAAGTTTCGAGCTGGACTCAGTG
gtwin13-FLAPX	TCGAAACGGCCTGGTAATGCCCTCCTAAGTTTCGAGCTGGACTCAGTG
gtwin14-FLAPX	TCGAAACGGCCTGGTAATGCCCTCCTAAGTTTCGAGCTGGACTCAGTG
gtwin15-FLAPX	TAGGACTGTGTTATGGGCAACCTCCTAAGTTTCGAGCTGGACTCAGTG
gtwin16-FLAPX	TGCAGTCTAGTCGAGCAATACCTCCTAAGTTTCGAGCTGGACTCAGTG
gtwin7-FLAPX	TAAGTACACGCAGCGACGTTCCTCCTAAGTTTCGAGCTGGACTCAGTG
gtwin18-FLAPX	TAAGTACACGCAGCGACGTTCCTCCTAAGTTTCGAGCTGGACTCAGTG
gtwin19-FLAPX	TATTGCATGAGGCTAAGCTCCCTCCTAAGTTTCGAGCTGGACTCAGTG
gtwin20-FLAPX	TGTCAACCGTTTCTCTACATCCTCCTAAGTTTCGAGCTGGACTCAGTG
gtwin21-FLAPX	GTGAGTCATAACTATCTTGTCCTCCTAAGTTTCGAGCTGGACTCAGTG
gtwin22-FLAPX	AAAGAATAGCACTGTCCGGTCCTCCTAAGTTTCGAGCTGGACTCAGTG
gtwin23-FLAPX	AGAGCATCCTCTCTAACTTCCCTCCTAAGTTTCGAGCTGGACTCAGTG
gtwin24-FLAPX	TTCCTTAGACCGGCAATGAACCTCCTAAGTTTCGAGCTGGACTCAGTG
gtwin25-FLAPX	GAGGGCAAATCCTTGGGTTGCCTCCTAAGTTTCGAGCTGGACTCAGTG
gtwin26-FLAPX	ATTCCCTAGCCAAAGCTAAACCTCCTAAGTTTCGAGCTGGACTCAGTG
gtwin27-FLAPX	GTTAGTGCGTTCTATGGTAGCCTCCTAAGTTTCGAGCTGGACTCAGTG

gtwin28-FLAPX	TAGAGCCTTTGCGTATGTAGCCTCCTAAGTTTCGAGCTGGACTCAGTG
gtwin29-FLAPX	TCATAGGTGATTGCCTTGTCCCTCCTAAGTTTCGAGCTGGACTCAGTG
gtwin30-FLAPX	TTGAGTGGTTCCGATCTTTTCCTCCTAAGTTTCGAGCTGGACTCAGTG
gtwin31-FLAPX	AAATGAGGGTTTTTCCCTTGCCTCCTAAGTTTCGAGCTGGACTCAGTG
gtwin32-FLAPX	TTATTTTGTCTACCCTGTTTCCTCCTAAGTTTCGAGCTGGACTCAGTG
gtwin33-FLAPX	TAGCTGTTATTATTCTGGGCCCTCCTAAGTTTCGAGCTGGACTCAGTG
gtwin34-FLAPX	TAGGCTCAGGTGACTGATTCCCTCCTAAGTTTCGAGCTGGACTCAGTG
gtwin35-FLAPX	GTTACAGTGGAGCCTAACTCCCTCCTAAGTTTCGAGCTGGACTCAGTG
gtwin36-FLAPX	AAGGAGGTCGGTTGCCTATGCCTCCTAAGTTTCGAGCTGGACTCAGTG
gtwin37-FLAPX	GAATCTCTTCTGGCCACGAGCCTCCTAAGTTTCGAGCTGGACTCAGTG
gtwin38-FLAPX	TGTTGGATGGTGTTCTGCACCCTCCTAAGTTTCGAGCTGGACTCAGTG
gtwin39-FLAPX	ATTCGCTGTCGTTTTTATCGCCTCCTAAGTTTCGAGCTGGACTCAGTG
gtwin40-FLAPX	CGTCATCTATCTCGGTAACGCCTCCTAAGTTTCGAGCTGGACTCAGTG
gtwin41-FLAPX	GCGCTCCCTAAAAAATTGATCCTCCTAAGTTTCGAGCTGGACTCAGTG
gtwin42-FLAPX	GTTCAATGAACGGCAGTCGGCCTCCTAAGTTTCGAGCTGGACTCAGTG
gtwin43-FLAPX	AATTTCCTCCCATGGAACTGCCTCCTAAGTTTCGAGCTGGACTCAGTG
zam1-FLAPY	ATTGCCAACATTTCTTTTTCTTTACACTCGGACCTCGTCGACATGCATT
zam2-FLAPY	ACGCCATAAATATATGGTCTTTACACTCGGACCTCGTCGACATGCATT
zam3-FLAPY	TTAGTGGCTTGTGATCTGTATTACACTCGGACCTCGTCGACATGCATT
zam4-FLAPY	GTTGGGTTCTTTGAAATTCATTTACACTCGGACCTCGTCGACATGCATT
zam5-FLAPY	TTACACTCGGACCTCGTCGACATGCATTGTCTCCAACGAATTATTTTT
zam6-FLAPY	TTTGTGAACCTTTCTTGTGATTACACTCGGACCTCGTCGACATGCATT
zam7-FLAPY	TTACACTCGGACCTCGTCGACATGCATTTTTAAATTTGGGTCCGCTCTAC
zam8-FLAPY	ATGTGGGGCAAGGCTTAACATTACACTCGGACCTCGTCGACATGCATT
zam9-FLAPY	TTACACTCGGACCTCGTCGACATGCATTTTCGTTAATAGGTTTTTCGG
zam10-FLAPY	TCTAGTATGAGTTGCGTGTTTTACACTCGGACCTCGTCGACATGCATT
zam11-FLAPY	GGTGTTGTAGTTTGGTAAGATTACACTCGGACCTCGTCGACATGCATT
zam12-FLAPY	TTACACTCGGACCTCGTCGACATGCATTGGCTGTGAATATTTTTTCCT
zam13-FLAPY	AGGGTTCCTTTCAAGATTTTTTACACTCGGACCTCGTCGACATGCATT
zam14-FLAPY	ATTATCGTCCGCCAAGAATGTTACACTCGGACCTCGTCGACATGCATT
zam15-FLAPY	GCTTTTTCGATTAATGCGGATTACACTCGGACCTCGTCGACATGCATT
zam16-FLAPY	TCCTACGATTTCGTGGAGAATTACACTCGGACCTCGTCGACATGCATT
zam17-FLAPY	TTACACTCGGACCTCGTCGACATGCATTTGAATTTGTTTTGTTCGGGGT
zam18-FLAPY	TGCTGTTAGTGTGATATTCCTTACACTCGGACCTCGTCGACATGCATT
zam19-FLAPY	GAATGTTTCGTCTATGCCTCTTACACTCGGACCTCGTCGACATGCATT
zam20-FLAPY	GTTTTTCATATTGGGGAAGTTTACACTCGGACCTCGTCGACATGCATT
zam21-FLAPY	AGGTTTCGCAATTCCTAATTTTACACTCGGACCTCGTCGACATGCATT
zam22-FLAPY	GTCGTATTTGAGTTTTAGACTTACACTCGGACCTCGTCGACATGCATT
zam23-FLAPY	GGGTTTCTGGGGTTTCAAATTTACACTCGGACCTCGTCGACATGCATT
zam24-FLAPY	GTATTATGTCGAGGGGTTTCTTACACTCGGACCTCGTCGACATGCATT
zam25-FLAPY	TTTGTCTATGATTGTCAGGTTTACACTCGGACCTCGTCGACATGCATT
zam26-FLAPY	TTTCTATTTGGGATGGGGTATTACACTCGGACCTCGTCGACATGCATT

zam27-FLAPY	TTACACTCGGACCTCGTCGACATGCATTCTTTGATGCAATTGATGCCG
zam28-FLAPY	GGTAGTCCGAATTGACTGAATTACACTCGGACCTCGTCGACATGCATT
zam29-FLAPY	TTACACTCGGACCTCGTCGACATGCATTTCGTTGCAAAATTCTACTCCT
zam30-FLAPY	GCTGGAAGGATGTGACATGGTTACACTCGGACCTCGTCGACATGCATT
zam31-FLAPY	CGTTCTACTGGAGAATTACTTTTACACTCGGACCTCGTCGACATGCATT
zam32-FLAPY	TCTCTGTCAAAGAGGAGTGTTTACACTCGGACCTCGTCGACATGCATT
zam33-FLAPY	TTTTCCGTGTGTCTAGTATTTTACACTCGGACCTCGTCGACATGCATT
zam34-FLAPY	TTCTGACATTATTTCTTCGTTTACACTCGGACCTCGTCGACATGCATT
zam35-FLAPY	GGAGTGGATTGCGTTGTTATTTACACTCGGACCTCGTCGACATGCATT
zam36-FLAPY	TTACACTCGGACCTCGTCGACATGCATTAAAAGGGGTGTGTTTGGTGG
zam37-FLAPY	TTACACTCGGACCTCGTCGACATGCATTCGAATAAATGGGTCCTACCA
zam38-FLAPY	TTACACTCGGACCTCGTCGACATGCATTTCATGCTCATTATTGGGTAT

Supplementary Table S7: List of primers used for smiFISH experiments

		gtwin			ZAM		
	Proportion	Obs	Exp	P.adj	Obs	Exp	P.adj
Sci-ATAC-seq 0-2h	0.084992	49	17.85	9.25694e-11	9	8.58	0.857736
ATAC-seq PGC	0.017645	2	3.71	0.595270	5	1.78	0.134952
Overlapping	0.024339	27	5.11	5.67542e-12	3	2.46	0.857736
Other	0.873024	132	183.34	8.91007e-19	84	88.18	0.459302
Sci-ATAC-seq 0-2h	0.093613	58	19.66	6.59140e-14	10	9.45	0.863642
ATAC-seq PGC unique	0.010916	2	2.29	1	5	1.10	0.020664
Overlapping	0.015718	18	3.30	1.20960e-08	2	1.59	0.863642
Other	0.879753	132	184.75	3.44636e-20	84	88.86	0.599892

Supplementary Table S8: Distribution of gtwin and ZAM new insertions based on chromatin accessibility in 0-2h embryos (sci-ATAC-seq) and PGC of late embryos (ATAC-seq) at their respective landing sites. « Obs » and « Exp » refer to the observed and expected numbers of LTR-RTE insertions for each genomic and chromatin feature. Adjusted p-values ("P.adj") were calculated using binomial tests corrected with the Benjamini–Hochberg step-up procedure to control the false discovery rate.

		gtwin		
	Genomic Proportion	Obs	Exp	p-value
Opa ChIP-seq 2-4hrs AEL	0.048	22	10	5.775e-4
GAF ChIP-seq2-4hrs AEL	0.022	15	5	8.423e-5
Zelda ChIP-seq2-4hrs AEL	0.018	8	4	0.0607
Clamp ChIP-seq2-4hrs AEL	0.024	8	5	0.1799
Sci-ATAC-seq 2-4hrs AEL	0.085	76	18	< 2.2 e-16
Opa ChIP-seq alone	0.020	2	4	0.4528
GAF ChIP-seq alone	0.009	4	2	0.1269
Opa ChIP-seq in sci-ATAC-seq 2-4h	0.028	20	6	2.404e-6
GAF ChIP-seq in sci-ATAC-seq 2-4h	0.013	11	3	1.309e-4

Supplementary Table S9: Distribution of gtwin insertion sites based on Chip-seq datasets for pioneer factors listed in (Supplementary Table S6) and chromatin accessibility in 2-4 hrs embryos (sci-ATAC-seq) (37). « Obs » and « Exp » refer to the observed and expected numbers of gtwin insertions for each defined genomic region. p-values were calculated using binomial tests.
